# Association between serum tumor necrosis factor-alpha levels and rheumatoid arthritis-associated interstitial lung disease: a cross-sectional study

**DOI:** 10.3389/fimmu.2026.1881556

**Published:** 2026-08-26

**Authors:** Xiaoli Li, Lina Leng, Yaorong Han, Jinfeng Zhang, Xinfang Shang, Shusen Zhang, Dengxiang Liu, Yuhua Qiao

**Affiliations:** 1 Department of Rheumatology, Xingtai People’s Hospital, Xingtai, Hebei, China; 2 Hebei Province Xingtai People’s Hospital Postdoctoral Workstation, Xingtai, Hebei, China; 3 Department of Radiology, Xingtai People’s Hospital, Xingtai, Hebei, China; 4 Department of Pulmonary and Critical Care Medicine, Xingtai People’s Hospital, Xingtai, Hebei, China; 5 Hebei Provincial Key Laboratory of Portal Hypertension and Cirrhosis, Xingtai People’s Hospital, Xingtai, Hebei, China; 6 Department of Surgical Urology, Xingtai People’s Hospital, Xingtai, Hebei, China

**Keywords:** cross-sectional study, nonlinear association, rheumatoid arthritis, rheumatoid arthritis-associated interstitial lung disease, tumor necrosis factor-α

## Abstract

**Background:**

Rheumatoid arthritis-associated interstitial lung disease (RA-ILD) is one of the most severe extra-articular complications of rheumatoid arthritis (RA). Although tumor necrosis factor-α (TNF-α) plays a central role in RA pathogenesis, its relationship with RA-ILD remains uncertain. This study aimed to investigate the association between serum TNF-α levels and RA-ILD, with a focus on sex-stratified patterns.

**Methods:**

We performed a cross-sectional study that initially included 1,191 consecutive RA inpatients at Xingtai People’s Hospital between March 2022 and December 2024. Clinical and laboratory data were extracted from electronic medical records. RA-ILD was diagnosed using high-resolution computed tomography, with subtypes classified by consensus. Multivariable logistic regression and generalized additive models were used to assess the association of TNF-α with RA-ILD. Serum TNF-α was natural log-transformed (ln) to improve linearity and reduce the influence of extreme values.

**Results:**

After exclusions, 790 patients were included in the final analysis, of whom 149 (18.86%) had RA-ILD, with a higher prevalence in males than in females (30.68% *vs* 15.47%). In sex-stratified analyses, after adjusting for age, disease duration, rheumatoid factor, and anti-citrullinated protein antibody in both sexes, and additionally adjusting for smoking in males, serum TNF-α was associated with RA-ILD in females (highest *vs* lowest tertile: OR = 1.96, 95% CI: 1.10 – 3.48; *P* for trend < 0.01). This association followed a nonlinear pattern, with an exploratory inflection point above which elevated TNF-α showed a positive association with increased odds of RA-ILD. In males, no statistically significant association was observed across all models. However, the formal sex ×ln(TNF-α) interaction term was not statistically significant (*P* = 0.08).

**Conclusion:**

In sex-stratified analyses, serum TNF-α was associated with RA-ILD in females, exhibiting a nonlinear relationship with a potential inflection point; no statistically significant association was observed in males. However, as the interaction testing did not confirm a statistically significant sex difference, these findings are exploratory and warrant further prospective validation in larger cohorts.

## 1 Introduction

Rheumatoid arthritis (RA) is a chronic systemic autoimmune disorder affecting approximately 0.25–1.0% of the global population (1, 2), characterized by a pronounced female predominance with a female-to-male ratio of 2–3:1 (3). The disease represents one of the most severe complications, with prevalence estimates ranging from 10% to 30% depending on diagnostic modalities and population characteristics (4). Notably, rheumatoid arthritis-associated interstitial lung disease (RA-ILD) has emerged as a leading cause of death in RA patients, carrying a mortality rate of 10–20% and a median survival of merely 5–8 years post-diagnosis (5).

To date, several clinical, serological, and genetic risk factors for RA-ILD have been well characterized. Advanced age, male sex, prolonged disease duration, high cumulative inflammatory burden, smoking history, and positivity for rheumatoid factor (RF) and anti-citrullinated protein antibody (ACPA) are established determinants of RA-ILD susceptibility (6–8). However, the association between circulating cytokine biomarkers and RA-ILD remains underexplored. Tumor necrosis factor-α (TNF-α) is a pivotal proinflammatory cytokine in RA pathogenesis, primarily secreted by activated macrophages, T cells, and synovial fibroblasts (9–11). It drives synovial proliferation, leukocyte recruitment, and osteoclast activation, thereby orchestrating local inflammation and articular destruction (9, 10, 12–14). Beyond its articular effects, TNF-α contributes to extra-articular manifestations, particularly pulmonary complications, by mediating epithelial injury, fibroblast activation, and dysregulated extracellular matrix remodeling (15–17). While elevated TNF-α levels in serum and synovial fluid correlate with disease activity and joint damage, and anti-TNF biologics constitute a cornerstone of RA management (1, 18), the association between serum TNF-α levels and RA-ILD remains unclear. An early small-sample study by Jian et al. observed elevated serum TNF-α levels in RA-ILD patients compared with RA patients without ILD (19); however, larger sample studies with detailed subgroup analyses are lacking.

The relationship between TNF-α signaling and RA-ILD is complex and remains a subject of debate (5, 20–23). Early case reports and observational studies raised concerns that TNF inhibitors might induce *de novo* ILD or exacerbate pre-existing lung disease (21, 22), whereas recent systematic reviews and meta-analyses suggest a more heterogeneous safety profile (20). These conflicting findings underscore the context-dependent role of TNF-α in lung pathology. Importantly, existing literature has predominantly focused on the therapeutic impact of anti-TNF agents on ILD progression, leaving the association between serum TNF-α levels and RA-ILD largely uninvestigated.

Sex differences represent another critical, yet often overlooked, dimension of RA-ILD pathogenesis. Although RA is more prevalent in women, male sex is consistently associated with a higher risk of developing RA-ILD and poorer pulmonary outcomes (7). Therefore, the present cross-sectional study was designed to investigate the association between serum TNF-α levels and RA-ILD, with a particular focus on sex-stratified patterns.

## 2 Materials and methods

### 2.1 Study population

**Figure 1:**
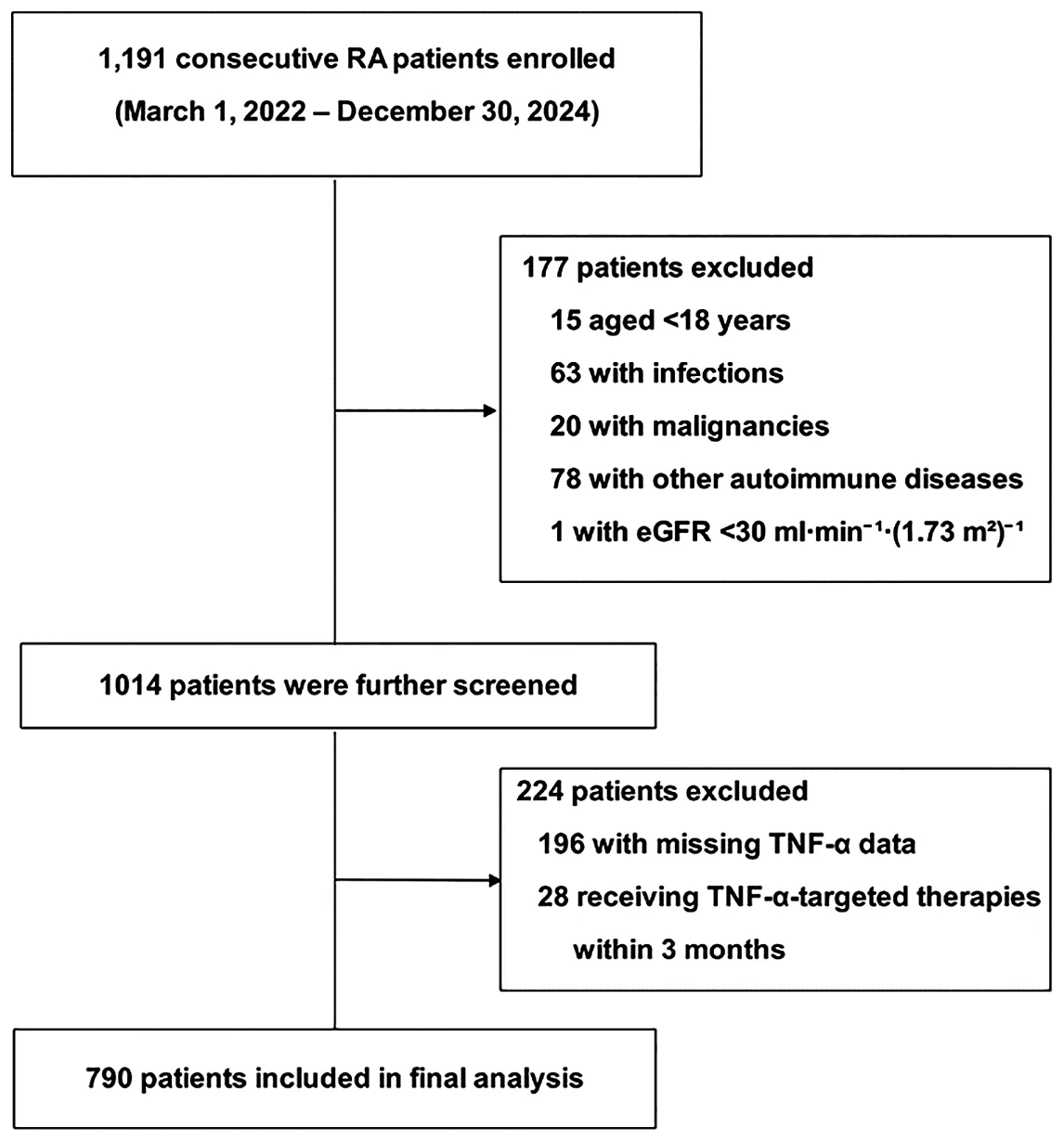
Flow diagram of patient screening and enrollment. eGFR, estimated glomerular filtration rate; RA, rheumatoid arthritis; TNF-α, tumor necrosis factor α.

This retrospective cross-sectional study included a consecutive cohort of 1,191 RA inpatients at Xingtai People’s Hospital from March 2022 to December 2024. All participants fulfilled the 2010 ACR/EULAR classification criteria for RA (24). Exclusion criteria included age <18 years, active or chronic infections, malignancies, other autoimmune diseases, severe renal dysfunction, treatment with TNF-α-targeted therapies within the prior three months, and absence of serum TNF-α data. Patients with missing TNF-α measurements did not differ significantly from those included in terms of clinical characteristics (**Supplementary Table 1**). Following these exclusions, 790 patients were eligible for analysis. The enrollment process is depicted in **Figure 1**. The study was conducted in accordance with the Declaration of Helsinki and approved by the Ethics Committee of Xingtai People’s Hospital (approval number: 2025[031]). Owing to its retrospective design and anonymization of patient data, informed consent was waived by the Ethics Committee.

### 2.2 Clinical data collection

Demographic and clinical variables were collected, including age, sex, body mass index (BMI), smoking status, alcohol consumption, and RA-related data comprised disease duration, current medication use [nonsteroidal anti-inflammatory drugs (NSAIDs), glucocorticoids, conventional synthetic disease-modifying antirheumatic drugs (csDMARDs), targeted synthetic DMARDs (tsDMARDs), and biologic DMARDs (bDMARDs)], swollen joint count, tender joint count, patient global assessment, and then disease activity score in 28 joints using the erythrocyte sedimentation rate (DAS28-ESR) was calculated. All clinical data were extracted from the hospital’s electronic medical record system and were originally obtained at admission through standardized interviews and physical examinations conducted by rheumatologists.

Smoking exposure was refined as smoking status (never/past/current), daily cigarette consumption (cigarettes/day), total smoking duration (years), and pack-years calculated as (cigarettes/day ÷ 20) × years. Heavy alcohol use was defined as documented excessive alcohol consumption in the medical records. Medication exposure was quantified for glucocorticoids, methotrexate (MTX), leflunomide (LEF), bDMARDs, and tsDMARDs. For glucocorticoids: we recorded (1) use within the past year (yes/no) (2); cumulative duration of use (months); and (3) average daily dose (mg/d). A cumulative exposure variable (mg·month) was then calculated as average daily dose × cumulative duration of use, reflecting the total glucocorticoid burden. For MTX, LEF, other bDMARDs and tsDMARDs: we recorded (1) use within the past year (yes/no); and (2) cumulative duration of use (months). For TNF-α inhibitors, because serum TNF-α is the primary exposure of interest, patients who had received TNF-α inhibitor therapy within the preceding 3 months were excluded from the main analysis. In sensitivity analyses, we further extended this exclusion window to 6 months and 12 months to assess the robustness of our findings.

Laboratory parameters, including erythrocyte sedimentation rate (ESR), C-reactive protein (CRP), RF, TNF-α, interleukin-6 (IL-6), and ACPA, were retrieved from the electronic database. Serum TNF-α concentrations were measured using a magnetic particle chemiluminescence immunoassay (Nanjing Aituo Biotechnology Co., Ltd., China) on an automated immunoassay analyzer, with a lower limit of detection of 2.5 pg/mL. Values below this threshold were assigned a value of 1 pg/mL prior to natural logarithmic transformation to avoid undefined values. Given approximately 34-month enrollment period, samples were assayed in multiple batches, with the reagent lot number changing approximately every two to three months. The intra-assay coefficient of variation ranged from 1.69% to 2.48%, and the inter-assay coefficient of variation ranged from 3.20% to 3.73%, based on routine quality control data from the central laboratory. The kit is registered with the National Medical Products Administration (NMPA) of China under Registration No. 20232400247, and is calibrated against the WHO International Standard for TNF-α. The manufacturer’s reference range in healthy adults is ≤5.5 pg/mL. All measurements were performed in the central laboratory under standardized protocols using fasting peripheral venous blood samples collected on the day of admission or the following morning.

### 2.3 High-resolution CT of the chest and RA-ILD subtype classification

All study participants underwent high-resolution computed tomography (HRCT) of the chest, with imaging data systematically extracted from medical records. HRCT was performed on the day of admission or within 48 hours thereafter, and blood samples for TNF-α measurement were collected on the same day or the following morning; thus, the time interval between HRCT and blood sampling was consistently within 48 hours for nearly all patients. Besides, HRCT and TNF-α measurements were routinely performed for all hospitalized RA patients, unless declined by the patient for personal or financial reasons.

The diagnosis of ILD in patients with RA was confirmed via chest HRCT. For RA-ILD subtype classification, we focused on the two most common subtypes, usual interstitial pneumonia (UIP) and nonspecific interstitial pneumonia (NSIP), and a consensus-based approach was employed. Subtype classification was based on standardized radiologic criteria (25): UIP was defined by a subpleural, basal-predominant distribution of reticular opacities with associated honeycombing; NSIP was characterized by bilateral ground-glass opacities, reticular abnormalities, and decreased lower lobe lung volumes. Cases with indeterminate patterns, organizing pneumonia, or lymphocytic interstitial pneumonia were not classified as RA-ILD for the primary analysis. For patients with mixed features of both UIP and NSIP, the pattern was assigned to UIP, as UIP is the more clinically aggressive phenotype and the predominant pattern in such mixed cases.

All HRCT images were independently reviewed by one experienced radiologist with 15 years of experience and one pulmonologist with 12 years of experience. The reviewers were blinded to all clinical information, including TNF-α levels, disease activity, and medication use; however, they were not blinded to sex, as this is inherent to imaging interpretation. In cases of disagreement, a senior rheumatologist adjudicated, with final diagnoses reached by consensus discussion.

### 2.4 Statistical analysis

Continuous variables were expressed as mean ± standard deviation (SD) or median (interquartile range), with group differences assessed using ANOVA or Kruskal-Wallis tests, as appropriate. Categorical variables were presented as numbers (percentages) and compared using χ² tests or Fisher’s exact test when expected frequencies were <5. Given the low proportion of missing data (<1% for all variables), we performed complete-case analyses without imputation, with the effective sample size reported for each model. Details on missing data are provided in **Supplementary Table 2**. Multicollinearity among inflammatory markers was assessed using variance inflation factors (VIF), with a threshold of VIF ≥ 5 indicating substantial collinearity (**Supplementary Table 3**).

Serum TNF-α was natural log-transformed (ln) for regression analyses to improve linearity with the logit of the outcome and to reduce the influence of extreme values, given its wide and right-skewed distribution. To enhance clinical interpretability, TNF-α was also analyzed in two additional ways: (1) as a standardized Z-score based on raw values, corresponding to a 1-SD increase in the original TNF-α distribution; and (2) as tertiles based on raw values, with the median value of each tertile assigned as a continuous variable for trend testing. Tertile cutoffs were reported in pg/mL for clinical readability (shown in **Supplementary Table 4**).

Covariate selection was guided by clinical plausibility and established risk factors (6–8). Variables were classified *a priori* as: baseline confounders (age, smoking, disease duration, RF, ACPA) included in the core model; inflammatory markers (ESR, IL-6) as potential mediators added in sensitivity analysis; and medication variables (glucocorticoids, MTX, LEF, other bDMARDs/tsDMARDs) as treatment-related factors added in sensitivity analysis with sex-specific selections to avoid collider bias. To avoid overfitting given limited events (54 in males, 95 in females), we adopted a parallel modelling strategy. All models shared a core adjustment set (age and disease duration in both sexes; smoking pack-years in males only), with each model adding one covariate block independently. Model I was adjusted for age and disease duration in both sexes, with additional adjustment for smoking (pack-year) in males. Model II was adjusted for Model I covariates plus autoantibodies (RF and ACPA in both sexes). Model III was adjusted for Model I covariates plus inflammatory markers (ESR and IL-6 in both sexes). Model IV was adjusted for Model I covariates plus cumulative exposure duration, with sex-specific selections strictly guided by event counts to preserve model stability. In males (54 events), we adjusted only for cumulative exposure duration of LEF and other bDMARDs/JAK inhibitors, excluding MTX and glucocorticoids to avoid collider bias and overfitting. In females (95 events), we adjusted for cumulative exposure duration of MTX, LEF, and other bDMARDs/tsDMARDs, and cumulative glucocorticoids exposure (mg·month).

Model 2 was selected as the primary model for inference, as RF and ACPA are established baseline confounders not influenced by ILD status; Models 3 and 4 served as sensitivity analyses. A formal sex × ln(TNF-α) interaction term was included in Model 2. Generalized additive models were employed to explore potential nonlinear relationships in Model 2. To explore potential threshold effects, we applied a two-piecewise logistic regression model with an inflection point estimated using a data-driven algorithm that maximizes model likelihood; robustness was assessed via bootstrap and restricted cubic splines.

All statistical analyses were conducted using R software (http://www.r-project.org, The R Foundation) and EmpowerStats (http://www.empowerstats.com, X&Y Solutions, Inc., Boston, MA). Statistical significance was defined as a two-sided *P* value < 0.05.

## 3 Results

### 3.1 Characteristics of study population

The study enrolled a total of 790 patients with RA, among whom 149 (18.86%) were diagnosed with RA-ILD. As detailed in **Table 1**, patients with RA-ILD were significantly older and had a higher proportion of males compared to those without RA-ILD. Inflammatory markers, including ESR, CRP, RF, and DAS28-ESR, were notably elevated in the RA-ILD group. Serum levels of IL-6 and TNF-α were also significantly higher in patients with RA-ILD. Regarding therapeutic regimens, glucocorticoid use was more prevalent in the RA-ILD, whereas the proportions of patients using MTX or LEF within the past year were lower. No statistically significant differences were observed in the use of NSAIDs or other bDMARDs/tsDMARDs between the two groups.

**Table 1:** Clinical characteristics of RA patients stratified by RA-ILD (N = 790).

Characteristics	RA-ILD		*P* value
	No	Yes	
N	641	149	
Sex			<0.01
Female	519 (80.97%)	95 (63.76%)	
Male	122 (19.03%)	54 (36.24%)	
Age (year)	56.38 (13.13)	64.76 (9.18)	<0.001
BMI (kg/m^2^)	23.94 (3.55)	24.30 (3.56)	0.273
DD (month)	48.00 (12.00-120.00)	60.00 (12.00-164.00)	0.28
Smoking, n (%)			0.21
No	582 (90.80%)	129 (86.58%)	
Yes	38 (5.93%)	11 (7.38%)	
Ever	21 (3.28%)	9 (6.04%)	
Smoking (pack-year)	0.00 (0.00-0.00)	0.00 (0.00-0.00)	0.03
Heavy alcohol use, n (%)	10 (1.56%)	4 (2.68%)	0.35
ESR (mm/H)	54.50 (31.00-86.00)	69.00 (42.00-90.00)	<0.01
CRP (mg/L)	22.66 (6.27-47.50)	34.94 (11.15-56.72)	<0.01
RF (IU/mL)	155.80 (44.60-327.60)	223.40 (66.70-399.30)	<0.01
ACPA (AU/mL)	200.00 (84.75-200.00)	200.00 (129.25-200.00)	0.04
DSA28ESR	5.08 (0.98)	5.33 (1.00)	<0.01
IL-6 (pg/mL)	35.05 (12.23-84.11)	48.44 (20.31-93.72)	<0.01
TNF-α (pg/mL)	9.43 (2.90-22.67)	13.80 (3.78-50.88)	<0.01
NSAIDs, n (%)	329 (51.33%)	77 (51.68%)	0.94
GCs use in past year, n (%)	68 (10.61%)	30 (20.13%)	<0.01
Cumulative GCs exposure (month)	0.00 (0.00-0.00)	0.00 (0.00-0.00)	0.02
MTX use in past year, n (%)	154 (24.02%)	23 (15.44%)	0.02
Cumulative MTX exposure duration (month)	0.00 (0.00-6.00)	0.00 (0.00-0.00)	0.02
LEF use in past year, n (%)	137 (21.37%)	23 (15.44%)	0.10
Cumulative LEF exposure duration (month)	0.00 (0.00-1.00)	0.00 (0.00-0.00)	0.03
Other bDMARDs or tsDMARDs, n (%)	24 (3.74%)	11 (7.38%)	0.05
Cumulative Other bDMARDs or tsDMARDs duration (month)	0.00 (0.00-0.00)	0.00 (0.00-0.00)	0.15

Values are presented as mean (SD), median (Q1–Q3) or n (%).

Note 1: bDMARDs exclude TNF-α inhibitors.

Note 2: Values for cumulative exposure duration are presented as median (Q1–Q3); the median of 0 (0–0) reflects that more than 75% of patients had no recorded exposure to the respective medication.

Note 3: The number of patients included in each variable using complete-case analysis.

RA-ILD, rheumatoid arthritis associated interstitial lung disease; BMI, body mass index; DD, disease duration; ESR, erythrocyte sedimentation rate; CRP, C-reactive protein; RF, rheumatoid factor; ACPA, anti-citrullinated protein antibody; DAS28ESR, disease activity score 28 using erythrocyte sedimentation rate; IL-6, interleukin-6; TNF-α, tumor necrosis factor α; NSAIDs, non-steroidal anti-inflammatory drugs; GCs, glucocorticoids; MTX, methotrexate; LEF, leflunomide; bDMARDs, biological disease-modifying antirheumatic drugs; tsDMARDs, targeted synthetic disease-modifying antirheumatic drugs

Subgroup analyses stratified by sex revealed distinct clinical profiles between male (n = 176) and female (n = 614) patients (**Table 2**). The prevalence of RA-ILD was significantly higher in males than in females (30.68% vs. 15.47%, *P* < 0.01). Significant sex differences were observed in the distribution of HRCT patterns: UIP was more prevalent in males than in females (19.89% *vs.* 4.56% of all patients in each sex subgroup, *P* < 0.01), whereas the prevalence of NSIP was similar between sexes (10.80% *vs.* 10.91%, *P* = 0.96). Male patients were older, had higher smoking and drinking rates, and exhibited shorter disease durations but higher levels of inflammatory markers, including ESR, CRP, RF, IL-6, and DAS28-ESR (all *P* < 0.05). However, serum TNF-α levels did not differ significantly between sexes (*P* = 0.80). Regarding therapeutic regimens, the proportion of patients using MTX within the past year was significantly lower in males than in females (13.07% vs. 25.08%, *P* < 0.01), while the use of GCs, LEF, and other bDMARDs/tsDMARDs within the past year was comparable between sexes.

**Table 2:** Clinical characteristics of RA patients stratified by sex (N = 790).

Characteristics	Sex		*P* value
	Male	Female	
N	176	614	
Age (year)	62.72 (10.26)	56.60 (13.26)	<0.01
BMI (kg/m^2^)	24.06 (3.34)	23.99 (3.62)	0.83
DD (month)	48.00 (12.00-104.00)	60.00 (12.00-144.00)	0.03
Smoking, n (%)			<0.01
No	98 (55.68%)	613 (99.84%)	
Yes	49 (27.84%)	0 (0.00%)	
Ever	29 (16.48%)	1 (0.16%)	
Smoking (pack-year)	0.00 (0.00-18.12)	0.00 (0.00-0.00)	<0.01
Heavy alcohol use, n (%)	14 (7.95%)	0 (0.00%)	<0.01
ESR (mm/H)	66.00 (40.75-90.25)	54.00 (32.00-85.00)	0.03
CRP (mg/L)	42.10 (22.67-66.74)	19.56 (5.56-42.79)	<0.01
RF (IU/mL)	213.25 (54.87-419.10)	159.65 (45.00-315.53)	0.01
ACPA (AU/mL)	200.00 (104.50-200.00)	200.00 (91.00-200.00)	0.29
DAS28ESR	5.31 (0.95)	5.07 (0.99)	<0.01
IL-6 (pg/mL)	57.92 (25.98-115.13)	33.38 (11.15-78.32)	<0.01
TNF-α (pg/mL)	10.96 (2.68-26.54)	9.81 (3.12-24.87)	0.80
GCs use in past year, n (%)	23 (13.07%)	75 (12.21%)	0.76
Cumulative GCs exposure (month)	0.00 (0.00-0.00)	0.00 (0.00-0.00)	0.47
MTX use in past year, n (%)	23 (13.07%)	154 (25.08%)	<0.01
Cumulative MTX exposure duration (month)	0.00 (0.00-0.00)	0.00 (0.00-6.00)	<0.01
LEF use in past year, n (%)	36 (20.45%)	124 (20.20%)	0.94
Cumulative LEF exposure duration (month)	0.00 (0.00-0.00)	0.00 (0.00-0.88)	0.44
Other bDMARDs or tsDMARDs, n (%)	5 (2.84%)	30 (4.89%)	0.25
Cumulative Other bDMARDs or tsDMARDs duration (month)	0.00 (0.00-0.00)	0.00 (0.00-0.00)	0.25
RA-ILD, n (%)	54 (30.68%)	95 (15.47%)	<0.01
NSIP, n (%)	19 (10.80%)	67 (10.91%)	0.96
UIP, n (%)	35 (19.89%)	28 (4.56%)	<0.01

Values are presented as mean (SD), median (Q1–Q3) or n (%).

Note 1: UIP and NSIP percentages are calculated using all patients in each sex subgroup.

Note 2: bDMARDs exclude anti-TNF-α inhibitors.

Note 3: Values for cumulative exposure duration are presented as median (Q1–Q3); the median of 0 (0–0) reflects that more than 75% of patients had no recorded exposure to the respective medication.

Note 4: The number of patients included in each variable using complete-case analysis.

BMI, body mass index; DD, disease duration; ESR, erythrocyte sedimentation rate; CRP, C-reactive protein; RF, rheumatoid factor; ACPA, anti-citrullinated protein antibody; DAS28ESR, disease activity score 28 using erythrocyte sedimentation rate; IL-6, interleukin-6; TNF-α, tumor necrosis factor α; GCs, glucocorticoids; MTX, methotrexate; LEF, leflunomide; NSAIDs, non-steroidal anti-inflammatory drugs; bDMARDs, biological disease-modifying antirheumatic drugs; tsDMARDs, targeted synthetic disease-modifying antirheumatic drugs; RA-ILD, rheumatoid arthritis associated interstitial lung disease; NSIP, nonspecific interstitial pneumonia; UIP, usual interstitial pneumonia.

These findings were further characterized by TNF-α tertile stratification. In females, the highest tertile was associated with significantly higher levels of CRP, IL-6, RF and ACPA, as well as a higher proportion of RA-ILD (**Supplementary Table 4**). In males, the highest tertile was associated with higher levels of IL-6 and RF (**Supplementary Table 5**).

### 3.2 Association between TNF-α and RA-ILD

**Table 3** presents the results of the sex-stratified multivariable logistic regression analysis examining the association between serumTNF-α levels and RA-ILD. In females, elevated TNF-α levels were associated with increased odds of RA-ILD across all models. In the primary model (Model 2), which adjusted for age, disease duration, RF, and ACPA in both sexes, and additionally adjusting for smoking (pack-year) in males, each 1-unit increase in ln(TNF-α) was associated with a 31% increase in the odds of RA-ILD in females (OR = 1.31, 95% CI: 1.09 - 1.58). When analyzed as a continuous variable standardized to a 1-SD increase based on raw TNF-α values, the OR was 1.33 (95% CI: 1.10 - 1.61). In the tertile analysis, female patients in the highest tertile had significantly higher odds of RA-ILD compared with those in the lowest tertile (OR = 1.96, 95% CI: 1.10 - 3.48; *P* for trend = 0.02). This association remained directionally consistent in sensitivity analyses further adjusting for inflammatory markers (Model 3) and cumulative medication exposures (Model 4), supporting the robustness of the findings. In males, no statistically significant association was observed across all models. However, the sex interaction was not statistically significant (*P* = 0.08, **Supplementary Table 6**).

**Table 3:** Multivariate logistic regressions results of association between TNF-α and RA-ILD.

Exposure	Model I OR (95% CI)	Model II OR (95% CI)	Model III OR (95% CI)	Model IV OR (95% CI)
Male				
N (Events)	176 (54)	175 (54)	176 (54)	176 (54)
ln(TNF-α) (per 1-unit)	1.05 (0.83, 1.32)	1.02 (0.80, 1.30)	1.03 (0.81, 1.31)	1.06 (0.83, 1.34)
TNF-α (per 1-SD)	1.16 (0.85, 1.58)	1.13 (0.82, 1.56)	1.13 (0.82, 1.55)	1.17 (0.85, 1.61)
TNF-α Tertiles				
Low	*Ref*	*Ref*	*Ref*	*Ref*
Middle	1.07 (0.48, 2.39)	1.09 (0.48, 2.44)	1.01 (0.44, 2.30)	1.06 (0.47, 2.37)
High	0.97 (0.43, 2.18)	0.90 (0.40, 2.06)	0.91 (0.40, 2.08)	0.97 (0.43, 2.21)
*P* for trend	0.95	0.82	0.83	0.95
Female				
N (Events)	614 (95)	603 (94)	611 (95)	614 (95)
ln(TNF-α) (per 1-unit)	1.39 (1.16, 1.66)	1.31 (1.09, 1.58)	1.37 (1.14, 1.64)	1.40 (1.16, 1.67)
TNF-α (per 1-SD)	1.40 (1.17, 1.69)	1.33 (1.10, 1.61)	1.40 (1.15, 1.70)	1.42 (1.18, 1.71)
TNF-α Tertiles				
Low	*Ref*	*Ref*	*Ref*	*Ref*
Middle	1.00 (0.54, 1.87)	1.02 (0.54, 1.91)	1.02 (0.54, 1.91)	1.05 (0.56, 1.97)
High	2.23 (1.27, 3.91)	1.96 (1.10, 3.48)	2.20 (1.25, 3.88)	2.32 (1.31, 4.09)
*P* for trend	< 0.01	0.02	< 0.01	< 0.01

Model I was adjusted for age and disease duration in both sexes, with additional adjustment for smoking (pack-year) in males.

Model II was adjusted for Model I covariates plus RF and ACPA.

Model III was adjusted for Model I covariates plus ESR and IL-6.

Model IV was adjusted for Model I covariates plus cumulative exposure duration of LEF and other bDMARDs/tsDMARDs in males, and cumulative GCs exposure, MTX, LEF, and other bDMARDs/tsDMARDs cumulative exposure duration in females.

The number of patients included in each model using complete-case analysis, and tertile cutoffs were derived from sex-specific distributions of TNF-α values.

TNF-α, tumor necrosis factor α; ln(TNF-α), natural logarithm of TNF-α; RA-ILD, rheumatoid arthritis associated interstitial lung disease; RF, rheumatoid factor; ACPA, anti-citrullinated protein antibody; ESR, erythrocyte sedimentation rate; IL-6, interleukin-6; MTX, methotrexate; LEF, leflunomide; bDMARDs, biological disease-modifying antirheumatic drugs; tsDMARDs, targeted synthetic disease-modifying antirheumatic drugs; GCs, glucocorticoids; OR, odds ratio; CI, confidence interval.

### 3.3 Non-linear association between TNF-α and RA-ILD in female patients

**Figure 2:**
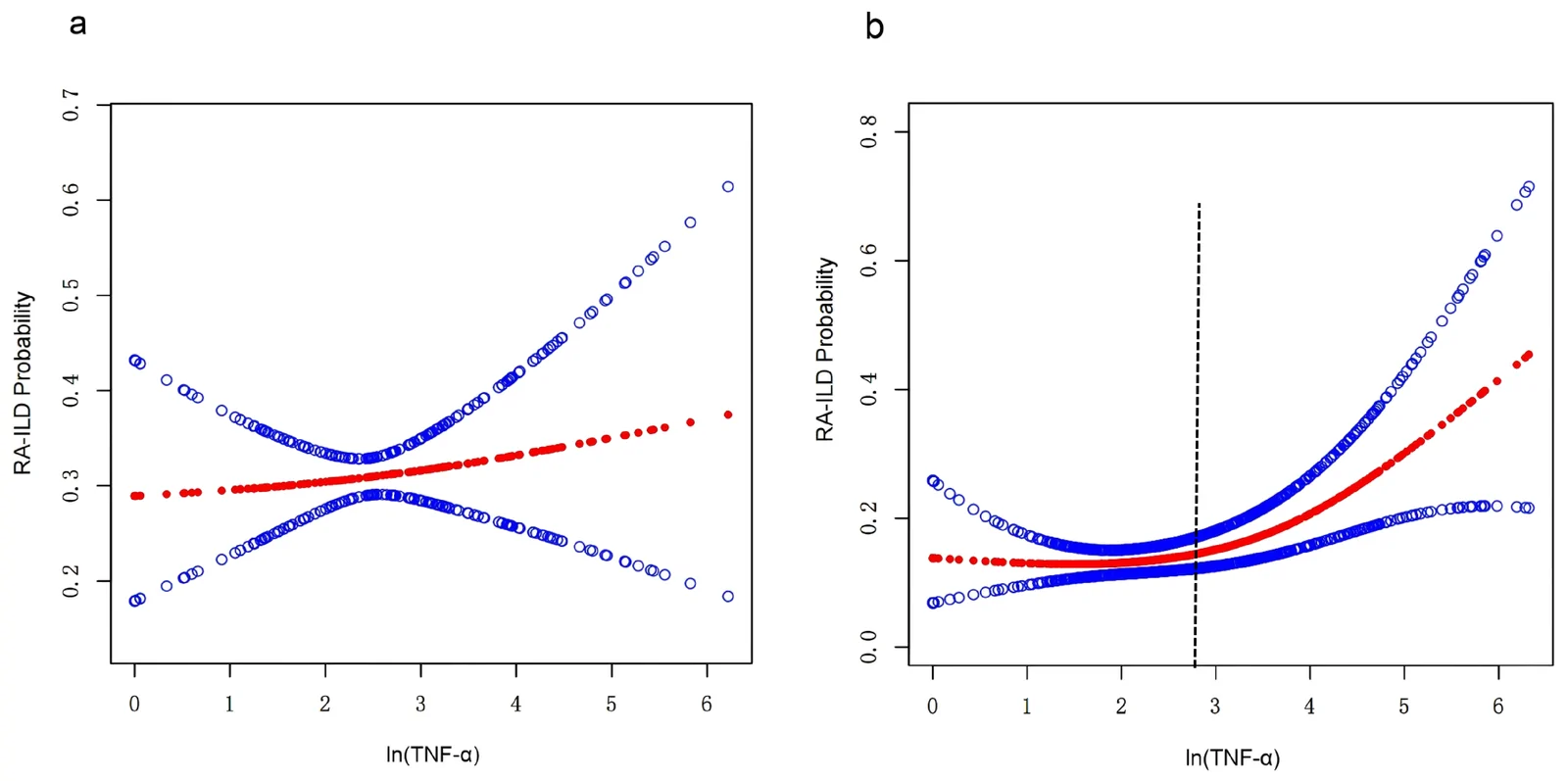
Sex-stratified smooth curve fitting of the association between serum TNF-α levels and prevalent RA-ILD. **(a)** male patients (n = 176); **(b)** female patients (n = 614). Solid lines represent the predicted probability of RA-ILD, and shaded areas indicate 95% confidence intervals. The vertical dashed line in **(b)** marks the inflection point at ln(TNF-α) = 2.74. The model was adjusted for age, disease duration, rheumatoid factor, and anti-citrullinated protein antibodies in both sexes, with additional adjustment for smoking (pack-year) in males. ln(TNF-α) natural logarithm of tumor necrosis factor α, RA-ILD rheumatoid arthritis-associated interstitial lung disease.

As illustrated in **Figure 2**, a nonlinear relationship between serum TNF-α levels and RA-ILD was observed in female patients after multivariable adjustment. To further evaluate this nonlinear pattern, a two-piecewise logistic regression model was performed. As detailed in **Table 4**, the analysis identified an exploratory inflection point at an ln(TNF-α) value of 2.74 (corresponding to a raw TNF-α concentration of approximately 15.46 pg/mL). Below this inflection point, TNF-α levels were not significantly associated with RA-ILD (OR = 0.94, 95% CI: 0.65–1.36, *P* = 0.75). Above the inflection point, however, elevated TNF-α levels showed a suggestive positive association with increased odds of RA-ILD (OR = 1.73, 95% CI: 1.24–2.40, *P* < 0.01). The log-likelihood ratio test *P* value was 0.04. Given the nature of this data-driven analysis and the marginal *P* value, this inflection point was exploratory and should be interpreted with caution.

**Table 4:** The results of two-piecewise logistic regression model of TNF-α with RA-ILD in female patients.

Model specification	N (Events)	RA-ILD OR (95%CI)	*P* value
Standard logistic regression	603 (94)	1.31 (1.09, 1.58)	< 0.01
Two-piecewise logistic regression			
Inflection point of ln(TNF-α) (95%CI): 2.74 (2.41, 3.20)			
≦2.74	378 (44)	0.94 (0.65, 1.36)	0.75
>2.74	225 (50)	1.73 (1.24, 2.40)	< 0.01
*P* for log-likelihood ratio test	0.04		

Note 1: The model was adjusted for age, disease duration, rheumatoid factor, anti-citrullinated protein antibodies.

Note 2: Ln TNF-α = 2.74 corresponds to a raw TNF-α concentration of approximately 15.46 pg/mL.

Note 3: N represents the number of patients included in each specific model based on complete-case analysis, and no imputation was performed for missing data.

ln(TNF-α), natural logarithm of tumor necrosis factor α; RA-ILD, rheumatoid arthritis associated interstitial lung disease; OR, odds ratio; CI, confidence interval. ORs represent the risk per 1-unit increase in ln(TNF-α). For the two-piecewise model, the effect was estimated separately below and above the inflection point.

### 3.4 Sensitivity analyses

To assess the robustness of our findings, we performed five sensitivity analyses. First, we extended the exclusion period for TNF-α inhibitor use to 6 months and 12 months prior to admission. The association between TNF-α and RA-ILD in females remained consistent with the main analysis (**Supplementary Table 7**). Second, we performed a sensitivity analysis excluding patients who had used bDMARDs or tsDMARDs within the past year. The association between TNF-α and RA-ILD remained consistent with the main analysis (**Supplementary Table 8**). Third, we excluded patients with a documented diagnosis of ILD prior to the index hospitalization (n = 34). The results remained essentially unchanged (**Supplementary Table 9**), suggesting that the observed associations were not substantially influenced by pre-existing ILD. Fourth, to validate the nonlinear pattern identified by the generalized additive model, we performed a sensitivity analysis using restricted cubic splines with the same covariates as in the main analysis. The restricted cubic spline model confirmed a similar nonlinear pattern in females, with a comparable transition point around the identified inflection point (**Supplementary Figure 1**). Fifth, to assess whether the definition of TNF-α tertiles influenced our findings, we performed a supplementary analysis using pooled tertiles based on the entire cohort distribution, rather than sex-specific cutoffs. The results remained consistent with the sex-specific tertile approach, confirming the robustness of our findings (**Supplementary Table 10**).

## 4 Discussion

In this cross-sectional study of 790 RA patients, the overall prevalence of RA-ILD was 18.86%, with a higher rate observed in males compared to females (30.68% *vs* 15.47%). In sex-stratified analyses, elevated TNF-α levels were associated with increased odds of RA-ILD in female patients, and a nonlinear relationship was identified with an exploratory inflection point at approximately 15.46 pg/mL, above which TNF-α showed a suggestive positive association with RA-ILD. No statistically significant association was observed in males. However, the formal interaction test was not statistically significant (*P* = 0.08), indicating that these sex-stratified patterns did not reach formal evidence of heterogeneity. Therefore, these sex-stratified findings should be considered exploratory and warrant further validation in larger prospective cohorts.

The reported prevalence of RA-ILD exhibits considerable heterogeneity across studies, primarily driven by disparities in demographic characteristics (e.g., sex, age, disease duration), variations in diagnostic modalities, and the rigor of screening protocols. While earlier studies reported a wide prevalence range of 2% to 58% (4, 26), more recent estimations utilizing standardized HRCT criteria suggest a refined prevalence of 10% to 30% in the general RA population (27–29), with approximately 5% to 10% presenting with clinically significant disease (4, 29–31). In the present study population of 790 Chinese RA patients, we identified an overall RA-ILD prevalence of 18.86% (n=149), a finding that aligns closely with contemporary literature. Notably, this substantial prevalence underscores the critical need for vigilant pulmonary surveillance in RA management and highlights the critical need to screen for risk factors associated with RA-ILD pathogenesis.

In the present study, the prevalence of RA-ILD was markedly higher in male patients compared to females (30.68% *vs* 15.47%), corroborating prior observations that, despite the well-established female predominance in RA overall, male sex confers a disproportionately elevated risk for pulmonary complications (7). Besides, UIP was the predominant pattern among males with RA-ILD (35/54, 64.81%), whereas NSIP was more prevalent in females with RA-ILD (67/95, 70.53%). However, these findings are derived from descriptive subgroup analyses without adjustment for confounding factors. Therefore, they should be interpreted with caution and do not establish a definitive sex-based difference in radiographic phenotypes. A similar observation has also been reported by Lee et al. (32), who found a male predominance in UIP and a female predominance in NSIP; however, that study was limited by a small sample size (n=18), highlighting the need for larger prospective cohorts to further validate these findings. Notably, UIP and NSIP represent the most common subtypes of RA-ILD, with UIP widely recognized as a phenotype associated with more severe and progressive lung pathology (33–35). Recent research by Solomon et al. demonstrated that RA-ILD patients with UIP patterns experienced a more rapid decline in pulmonary function and higher mortality rates compared to those with NSIP patterns (44% vs 24%), along with a shorter median survival time (10.18 years vs 13.62 years). However, after adjusting for pulmonary physiology (FVC), the HRCT pattern was no longer an independent predictor of mortality, suggesting that physiological impairment, rather than the radiographic phenotype itself, drives outcomes (36). While the prognostic significance of UIP versus NSIP has been well studied, the mechanisms underlying sex-specific differences in radiographic phenotype distribution remain poorly understood. Future studies stratified by sex are warranted to better characterize these differences and to inform more individualized risk stratification and management strategies.

Despite comparable circulating TNF-α levels between sexes (*P* = 0.80), elevated serum TNF-α was associated with increased odds of RA-ILD in female patients; no such association was observed in males. However, the formal sex × ln(TNF-α) interaction term was not statistically significant (*P* = 0.08), indicating that the apparent sex-stratified pattern did not reach formal evidence of heterogeneity. The lack of association in males may reflect a true absence of effect, but it may also be partly attributable to limited statistical power, as the male subgroup contained only 54 RA-ILD events, which restricts the precision of estimates and the ability to detect modest effects. Whether this null finding in males represents a genuine biological difference or a power-related artifact requires further validation in larger, adequately powered cohorts.

The observed nonlinear association between serum TNF-α and RA-ILD in female patients emerged from empirical patterns noted in the multivariable logistic regression analyses (**Table 3**). A disproportionate increase in odds ratios across TNF-α tertiles suggested a potential nonlinear relationship, prompting further exploration. Traditional linear modelling may attenuate the true biological effect by averaging the null association below the inflection point with the positive association above it, which could explain the relatively modest effect sizes observed in the fully adjusted linear models. Based on these observations, we applied generalized additive models and two-piecewise logistic regression (**Table 4**, **Figure 2**), which revealed a nonlinear pattern with an exploratory inflection point at approximately 15.46 pg/mL. Notably, a suggestive positive association with RA-ILD was observed only above this level. However, the inflection point identified in this analysis should be interpreted with caution. It was derived from a single-center cohort using a data-driven recursive algorithm, and its value shifted slightly after adjustment for different covariate sets. Although bootstrap resampling and restricted cubic spline sensitivity analyses supported the presence of a nonlinear pattern, the exact location of the inflection point remained within a modest range rather than a fixed cut-off. Therefore, this finding should be considered exploratory rather than a clinically actionable threshold.

The mechanisms underlying these sex-stratified observations remain unclear. TNF-α is a well-established central pro-inflammatory mediator in RA pathogenesis, yet its role in RA-ILD remains unclear, particularly regarding whether sex-based differences exist. Wu et al. proposed a paradigm in which RA-ILD evolves temporally from early TNF-α-driven inflammatory changes (resembling NSIP) to later irreversible fibrotic disease (resembling UIP) (25). In our cohort, males predominantly exhibited the UIP pattern, while females more often showed the NSIP pattern. However, whether this UIP-predominant phenotype in males reflects a more rapid transition through the inflammatory phase, or an inherently different susceptibility to fibrotic pathways, cannot be determined from the present cross-sectional data. Experimental evidence from the TNF-Tg3647 mouse model demonstrated that female mice developed more severe and earlier-onset pulmonary vasculopathy and interstitial lung disease with markedly reduced survival compared with males, suggesting heightened female susceptibility to TNF-α-mediated lung pathology (37). This aligns with our finding that elevated serum TNF-α was associated with increased odds of RA-ILD in females. On the other hand, Poole et al. (38) showed in a collagen-induced arthritis (CIA) plus inhaled lipopolysaccharide (LPS) model that testosterone depletion attenuated lung injury, while testosterone replacement restored proinflammatory and profibrotic responses, suggesting that testosterone itself may promote pulmonary inflammation in males. This experimental observation is consistent with the higher RA-ILD prevalence observed in males in our cohort. However, the interplay between sex hormones, TNF-α signaling, and pulmonary fibrosis is inherently complex, and further mechanistic studies are needed to elucidate the underlying pathways.

It should also be noted that serum IL-6 levels were significantly higher in patients with RA-ILD than in those without, as shown in the baseline comparison (**Table 1**). However, IL-6 did not retain a significant association with RA-ILD after adjustment for confounders (results not shown). Therefore, the present study focused primarily on TNF-α, while the potential role of IL-6 warrants further investigation. Future longitudinal studies are required to further elucidate the interplay between sex, pulmonary inflammation, and fibrosis in RA-ILD.

Nevertheless, several limitations should be acknowledged. First, the cross-sectional design restricts causal inference; three competing possibilities cannot be distinguished: (a) TNF-α elevation contributing to RA-ILD, (b) subclinical ILD driving TNF-α elevation, or (c) a shared upstream factor driving both. While we excluded patients with pre-existing ILD (**Supplementary Table 9**), undiagnosed subclinical ILD cannot be fully ruled out. Second, serum TNF-α may not fully reflect local pulmonary inflammation, as invasive sampling was not feasible. Third, despite adjusting for major confounders including smoking pack-years in males, residual confounding from unmeasured environmental exposures or medications cannot be excluded, particularly passive smoking or household environmental exposures among females. Fourth, the radiological characterization relied on HRCT without systematic exclusion of other ILD etiologies or multidisciplinary discussion and pulmonary function tests, which may have introduced misclassification; moreover, our subtype analysis focused on UIP and NSIP due to the rarity of other patterns. Fifth, the nonlinear analysis was exploratory and data-driven; the inflection point requires external validation. Sixth, reagent lots for the TNF-α assay changed approximately every two to three months, but individual lot information was not recorded, precluding a formal batch-effect sensitivity analysis; this should be considered when interpreting the inflection point. Finally, the single-center, inpatient cohort limits generalizability to community-based or outpatient RA populations.

In conclusion, in this cross-sectional study of 790 RA patients, the overall prevalence of RA-ILD was 18.86%, with a higher prevalence in males than in females. In sex-stratified analyses, elevated serum TNF-α was associated with increased odds of RA-ILD in female patients, with a nonlinear pattern and an exploratory inflection point. No such association was observed in males. However, given the cross-sectional design and the non-significant interaction test, these findings should be interpreted with caution. Prospective longitudinal studies are warranted to validate these observations and to clarify the potential role of TNF-α in RA-ILD.

## Data Availability

The raw data supporting the conclusions of this article will be made available by the authors, without undue reservation.

## Supplementary material

Supplementary Figure 1 Restricted cubic spline analysis of the association between serum TNF-α levels and RA-ILD. **(a)** male patients (n = 176); **(b)** female patients (n = 614). The model was adjusted for age, disease duration, rheumatoid factor, and anti-citrullinated protein antibodies in both sexes, with additional adjustment for smoking (pack-year) in males. ln(TNF-α) natural logarithm of tumor necrosis factor α, RA-ILD rheumatoid arthritis-associated interstitial lung disease, OR odds ratio, CI confidence interval.
