# Supplementary material for: Association between serum tumor necrosis factor-alpha levels and rheumatoid arthritis-associated interstitial lung disease: a cross-sectional study
Source: Front Immunol. 2026 Aug 26;17:1881556. doi: 10.3389/fimmu.2026.1881556 (PMC13553761; doi:10.3389/fimmu.2026.1881556)
Supplement: Supplementary file 1 [file DataSheet1.pdf]

Supplementary Material

Supplementary Tables

Supplementary Table S1. Comparison of clinical characteristics between patients with and without TNF-α measurements (N=986)

TNF-α	Missing	Available	*P* value
N	196	790	
Female, n (%)	150 (76.53%)	614 (77.72%)	0.72
Age (year)	57.64 (12.19)	57.96 (12.90)	0.75
BMI (kg/m^2)	23.74 (3.30)	24.01 (3.55)	0.34
DD (month)	36.00 (8.75-141.00)	48.00 (12.00-132.00)	0.32
Smoking, n (%)	9 (4.59%)	49 (6.20%)	0.39
Heavy alcohol use, n (%)	5 (2.55%)	14 (1.77%)	0.48
ESR (mm/H)	58.00 (30.75-86.00)	56.00 (34.00-88.00)	0.52
CRP (mg/L)	27.82 (7.07-45.73)	25.47 (6.94-49.05)	0.68
RF (IU/mL)	105.25 (40.70-305.00)	168.50 (47.68-346.05)	0.24
ACPA, AU/ml	200.00 (66.00-200.00)	200.00 (93.00-200.00)	0.08
DSA28ESR	4.99 (0.95)	5.13 (0.99)	0.09
NSAIDs, n (%)	98 (50.00%)	406 (51.39%)	0.73
GCs use, n (%)	22 (11.22%)	98 (12.41%)	0.651
MTX use, n (%)	46 (23.47%)	177 (22.41%)	0.75
LEF use, n (%)	43 (21.94%)	160 (20.25%)	0.60

Other bDMARDs or tsDMARDs, n (%)	8 (4.08%)	35 (4.43%)	0.83
RA-ILD, n (%)	31 (15.82%)	149 (18.86%)	0.32

Note: As IL-6 and TNF-α were measured using the same multiplex assay kit, IL-6 data were also missing in patients without TNF-α measurements.

Abbreviations: TNF-α tumor necrosis factor α, BMI body mass index, DD disease duration, ESR erythrocyte sedimentation rate, CRP c-reactive protein, RF rheumatoid factor, ACPA anti-citrullinated protein antibody, DAS28ESR disease activity score 28 using erythrocyte sedimentation rate, NSAIDs non-steroidal anti-inflammatory drugs, GCs glucocorticoids, MTX methotrexate, LEF leflunomide, bDMARDs biological disease-modifying antirheumatic drugs, tsDMARDs targeted synthetic disease-modifying antirheumatic drugs, RA-ILD rheumatoid arthritis associated interstitial lung disease

Supplementary Table S2. Description of the missing variables

Variables	Female		Male	
	N (Missing data)	Events	N (Missing data)	Events
Age	614 (0)	95	176 (0)	54
BMI	610 (4)	95	176 (0)	54
Smoking	614 (0)	95	176 (0)	54
smoking pack-years	614 (0)	95	176 (0)	54
Heavy alcohol use, n (%)	614 (0)	95	176 (0)	54
DD	614 (0)	95	176 (0)	54
ESR	611(3)	95	176 (0)	54
CRP	610 (4)	95	176 (0)	54
RF	608 (6)	95	176 (0)	54
ACPA	609 (5)	94	175 (1)	54
DAS28ESR	611 (3)	95	176 (0)	54
IL-6	614 (0)	95	176 (0)	54
TNF-α	614 (0)	95	176 (0)	54
NSAIDs	614 (0)	95	176 (0)	54
Glu use in past year	614 (0)	95	176 (0)	54
Cumulative GLU exposure	614 (0)	95	176 (0)	54
MTX use in past year	614 (0)	95	176 (0)	54
Cumulative MTX exposure duration	614 (0)	95	176 (0)	54

LEF use in past year	614 (0)	95	176 (0)	54
Cumulative LEF exposure duration	614 (0)	95	176 (0)	54
Other bDMARDs or tsDMARDs	614 (0)	95	176 (0)	54
Cumulative Other bDMARDs or tsDMARDs duration	614 (0)	95	176 (0)	54
RA-ILD	614 (0)	95	176 (0)	54

Abbreviations: BMI body mass index, DD disease duration, ESR erythrocyte sedimentation rate, CRP c-reactive protein, RF rheumatoid factor, ACPA anti-citrullinated protein antibody, DAS28ESR disease activity score 28 using erythrocyte sedimentation rate, IL-6 interleukin-6, TNF-α tumor necrosis factor α, NSAIDs non-steroidal anti-inflammatory drugs, GCs glucocorticoids, MTX methotrexate, LEF leflunomide, bDMARDs biological disease-modifying antirheumatic drugs, tsDMARDs targeted synthetic disease-modifying antirheumatic drugs, RA-ILD rheumatoid arthritis associated interstitial lung disease

Supplementary Table S3. Multicollinearity assessment of inflammatory markers

Iteration	Variance Inflation Factor				
	ESR	CRP	RF	IL-6	TNF-α
**Female**	2.9	1.8	1,2	1.4	1.1
**Male**	1,7	1.8	1.1	1.2	1.1

Note: All variance inflation factor values were < 5, indicating no substantial multicollinearity.
Abbreviations: variance inflation factors (VIF), ESR erythrocyte sedimentation rate, CRP c-reactive protein, RF rheumatoid factor, IL-6 interleukin-6, TNF-α tumor necrosis factor α.

Supplementary Table S4. Clinical characteristics of RA patients stratified by TNF-α in females (N=614)

**Characteristics**	**TNF-α tertiles**			***P* value**
	**T1 (0, 00, 4.94)**	**T2 (4.96, 17.38)**	**T3 (17.42, 554.76)**	
N (events)	205 (23)	204 (24)	205 (48)	--
Age (year)	55.54 (13.37)	56.68 (12.97)	57.57 (13.41)	0.30
BMI (kg/m^2)	24.25 (3.76)	23.77 (3.36)	23.95 (3.71)	0.41
DD (month)	48.00 (9.00-120.00)	60.00 (12.00-144.00)	72.00 (12.00-168.00)	0.25
ESR (mm/H)	53.00 (34.00-89.00)	52.00 (29.00-84.00)	62.00 (35.00-85.00)	0.32
CRP (mg/L)	19.36 (4.34-46.95)	11.89 (4.66-36.41)	27.00 (7.38-47.12)	<0.01
RF (IU/mL)	152.40 (35.50-312.60)	79.95 (29.62-221.00)	231.40 (85.50-535.55)	<0.01
ACPA (AU/mL)	200.00 (56.00-200.00)	200.00 (74.00-200.00)	200.00 (146.50-200.00)	<0.01
DAS28ESR	5.07 (0.97)	5.00 (1.01)	5.15 (1.00)	0.31
IL-6 (pg/mL)	19.16 (4.81-54.47)	31.47 (11.20-71.25)	47.51 (25.19-106.43)	<0.01
TNF-α (pg/mL)	2.50 (2.50-3.11)	9.81 (7.43-12.73)	36.74 (25.02-77.49)	<0.01
NSAIDs, n (%)	109 (53.17%)	110 (53.92%)	98 (47.80%)	0.40
GCs use in past year, n (%)	24 (11.71%)	21 (10.29%)	30 (14.63%)	0.39
Cumulative GCs exposure (month)	0.00 (0.00-0.00)	0.00 (0.00-0.00)	0.00 (0.00-0.00)	0.14

MTX use in past year, n (%)	55 (26.83%)	53 (25.98%)	46 (22.44%)	0.55
Cumulative MTX exposure duration (month)	0.00 (0.00-4.00)	0.00 (0.00-8.00)	0.00 (0.00-9.00)	0.51
LEF use in past year, n (%)	35 (17.07%)	48 (23.53%)	41 (20.00%)	0.27
Cumulative LEF exposure duration (month)	0.00 (0.00-0.00)	0.00 (0.00-6.00)	0.00 (0.00-0.50)	0.05
Other bDMARDs or tsDMARDs, n (%)	9 (4.39%)	9 (4.41%)	12 (5.85%)	0.73
Cumulative Other bDMARDs or tsDMARDs duration (month)	0.00 (0.00-0.00)	0.00 (0.00-0.00)	0.00 (0.00-0.00)	0.92
RA-ILD, n (%)	23 (11.22%)	24 (11.76%)	48 (23.41%)	0.01

Values are presented as mean (SD), median (Q1–Q3) or n (%).
Note 1: bDMARDs exclude anti-TNF-α inhibitors.
Note 2: Values for cumulative exposure duration are presented as median (Q1–Q3); the median of 0 (0–0) reflects that more than 75% of patients had no recorded exposure to the respective medication.
Note 3: The number of patients included in each variable using complete-case analysis.
Abbreviations:BMI body mass index, DD disease duration, ESR erythrocyte sedimentation rate, CRP c-reactive protein, RF rheumatoid factor, ACPA anti-citrullinated protein antibody, DAS28ESR disease activity score 28 using erythrocyte sedimentation rate, IL-6 interleukin-6, TNF-α tumor necrosis factor α, NSAIDs non-steroidal anti-inflammatory drugs, GCs glucocorticoids, MTX methotrexate, LEF leflunomide, bDMARDs biological disease-modifying antirheumatic drugs, tsDMARDs targeted synthetic disease-modifying antirheumatic drugs, RA-ILD rheumatoid arthritis associated interstitial lung disease

Supplementary Table S5. Clinical characteristics of RA patients stratified by TNF-α in males (N=176)

Characteristics	TNF-α tertiles			*P* value
	T1 (0.00, 4.09)	T2 (4.24, 20.75)	T3 (21.07, 501.01)	
N	59 (18)	58 (19)	59 (17)	--
Age (year)	62.10 (11.57)	63.31 (10.48)	62.75 (8.66)	0.82
BMI (kg/m^2)	24.17 (3.07)	24.66 (3.55)	23.35 (3.32)	0.10
DD (month)	48.00 (9.00-108.00)	39.00 (7.25-84.00)	48.00 (12.00-100.00)	0.59
Smoking (pack-year)	0.00 (0.00-20.00)	0.00 (0.00-15.00)	0.00 (0.00-15.00)	0.83
Smoking, n (%)	22 (37.29%)	19 (32.76%)	8 (13.56%)	0.02
No	32 (54.24%)	30 (51.72%)	36 (61.02%)	
Yes	22 (37.29%)	19 (32.76%)	8 (13.56%)	
Ever	5 (8.47%)	9 (15.52%)	15 (25.42%)	
Drinking, n (%)	3 (5.08%)	9 (15.52%)	2 (3.39%)	0.03
ESR (mm/H)	65.00 (39.50-89.00)	68.00 (50.00-85.25)	64.00 (31.50-103.50)	0.76
CRP (mg/L)	36.54 (10.07-63.42)	42.80 (24.31-68.92)	48.98 (30.55-66.06)	0.25
RF (IU/mL)	214.30 (53.35-363.35)	141.00 (42.80-339.50)	284.00 (139.80-647.70)	0.01
ACPA (AU/mL)	200.00 (78.00-200.00)	200.00 (79.50-200.00)	200.00 (200.00-200.00)	0.05
DAS28ESR	5.19 (0.97)	5.41 (0.93)	5.32 (0.93)	0.43
IL-6 (pg/mL)	35.46 (10.32-68.55)	57.06 (29.62-125.23)	89.40 (53.58-122.77)	<0.01
TNF-α (pg/mL)	2.50 (0.68-2.62)	10.96 (7.34-14.29)	50.00 (27.04-84.75)	<0.01

NSAIDs, n (%)	36 (61.02%)	27 (46.55%)	26 (44.07%)	0.14
GCs use in past year, n(%)	9 (15.25%)	7 (12.07%)	7 (11.86%)	0.83
Cumulative GCs exposure (month)	0.00 (0.00-0.00)	0.00 (0.00-0.00)	0.00 (0.00-0.00)	0.60
MTX use in past year, n (%)	11 (18.64%)	5 (8.62%)	7 (11.86%)	0.26
Cumulative MTX exposure duration (month)	0.00 (0.00-0.00)	0.00 (0.00-0.00)	0.00 (0.00-2.00)	0.48
LEF use in past year, n (%)	12 (20.34%)	13 (22.41%)	11 (18.64%)	0.88
Cumulative LEF exposure duration (month)	0.00 (0.00-0.00)	0.00 (0.00-0.00)	0.00 (0.00-0.00)	0.98
Other bDMARDs or tsDMARDs, n (%)	2 (3.39%)	1 (1.72%)	2 (3.39%)	0.82
Cumulative Other bDMARDs or tsDMARDs duration (month)	0.00 (0.00-0.00)	0.00 (0.00-0.00)	0.00 (0.00-0.00)	0.84
RA-ILD, n (%)	18 (30.51%)	19 (32.76%)	17 (28.81%)	0.96

Values are presented as mean (SD), median (Q1–Q3) or n (%).
Note 1: bDMARDs exclude anti-TNF-α inhibitors.
Note 2: Values for cumulative exposure duration are presented as median (Q1–Q3); the median of 0 (0–0) reflects that more than 75% of patients had no recorded exposure to the respective medication.
Note 3: The number of patients included in each variable using complete-case analysis.
Abbreviations: BMI body mass index, DD disease duration, ESR erythrocyte sedimentation rate, CRP c-reactive protein, RF rheumatoid factor, ACPA anti-citrullinated protein antibody, DAS28ESR disease activity score 28 using erythrocyte sedimentation rate, IL-6 interleukin-6, TNF-α tumor necrosis factor α, NSAIDs non-steroidal anti-inflammatory drugs, GCs glucocorticoids, MTX methotrexate, LEF leflunomide, bDMARDs biological disease-modifying antirheumatic drugs, tsDMARDs targeted synthetic disease-modifying antirheumatic drugs, RA-ILD rheumatoid arthritis associated interstitial lung disease

Supplementary Table S6. Interaction testing between sex-TNF α for RA-ILD across adjustment models

Model	Male OR (95%CI)	Female OR (95%CI)	*P* for interaction
Crude Model	1.07 (0.85, 1.34)	1.42 (1.20, 1.69)	0.05
Model I	1.05 (0.83, 1.32)	1.39 (1.16, 1.66)	0.07
Model II	1.02 (0.80, 1.30)	1.31 (1.09, 1.58)	0.08
Model III	1.03 (0.81, 1.31)	1.37 (1.14, 1.64)	0.09
Model IV	1.06 (0.83, 1.34)	1.38 (1.15, 1.65)	0.08

Note 1: TNF-α was natural log-transformed prior to analysis.
Note 2: The number of patients included in each model using complete-case analysis.
Note 3: Crude Model was unadjusted; Model I was adjusted for age, disease duration and smoking (pack-year); Model II was adjusted for Model I covariates plus RF and ACPA; Model III was adjusted for Model I covariates plus ESR and IL-6. Model IV was adjusted for Model I covariates plus cumulative exposure duration of LEF and other bDMARDs/tsDMARDs.
Note 4: Data are presented as odds ratios (OR) with 95% confidence intervals (CI). *P* values for interaction are derived from the sex × ln (TNF-α) term added to each corresponding model.
Abbreviations: RF rheumatoid factor, ACPA anti-citrullinated protein antibody, ESR erythrocyte sedimentation rate, IL-6 interleukin-6, LEF leflunomide, bDMARDs biological disease-modifying antirheumatic drugs, tsDMARDs targeted synthetic disease-modifying antirheumatic drugs.

Supplementary Table S7. Sensitivity analysis for the association between serum TNF-α and RA-ILD in females after extending TNF inhibitor exclusion periods to 6 months and 12 months

Exposure	Model 1<br>OR (95%CI)	Model 2<br>OR (95%CI)	Model 3<br>OR (95%CI)	Model 4<br>OR (95%CI)
**Excluding TNFi use within 6 months**				
N (Events)	590 (73)	580 (72)	583 (73)	590 (73)
ln(TNF-α) (per 1-unit)	1.40 (1.17, 1.67)	1.32 (1.09, 1.59)	1.37 (1.14, 1.65)	1.39 (1.16, 1.67)
TNF-α (per 1-SD)	1.41 (1.17, 1.69)	1.33 (1.10, 1.61)	1.39 (1.14, 1.69)	1.41 (1.17, 1.70)
TNF-α Tertiles				
Low	*Ref*	*Ref*	*Ref*	*Ref*
Middle	1.05 (0.56, 1.97)	1.06 (0.57, 2.00)	1.04 (0.56, 1.96)	1.02 (0.54, 1.94)
High	2.28 (1.29, 4.02)	1.99 (1.11, 3.57)	2.22 (1.25, 3.95)	2.25 (1.26, 4.00)
P for trend	< 0.01	0.02	< 0.01	< 0.01
**Excluding TNFi use within 12 months**				
N (Events)	587 (73)	577 (72)	580 (73)	587 (73)
ln(TNF-α) (per 1-unit)	1.40 (1.16, 1.67)	1.32 (1.09, 1.59)	1.37 (1.14, 1.65)	1.39 (1.16, 1.67)
TNF-α (per 1-SD)	1.40 (1.17, 1.69)	1.33 (1.10, 1.61)	1.39 (1.14, 1.69)	1.41 (1.17, 1.69)
TNF-α Tertiles				
Low	*Ref*	*Ref*	*Ref*	*Ref*
Middle	1.05 (0.56, 1.96)	1.06 (0.56, 1.99)	1.04 (0.55, 1.96)	1.01 (0.53, 1.93)
High	2.28 (1.29, 4.03)	1.99 (1.11, 3.58)	2.23 (1.25, 3.97)	2.25 (1.27, 4.01)

P for trend	< 0.01	0.02	< 0.01	< 0.01

Model I was adjusted for age and disease duration;
Model II was adjusted for Model I covariates plus RF and ACPA;
Model III was adjusted for Model I covariates plus ESR and IL-6;
Model IV was adjusted for Model I covariates plus cumulative GCs exposure, MTX, LEF, and other bDMARDs/tsDMARDs exposure duration.

Note: The number of patients included in each model using complete-case analysis.

Abbreviations: TNF-α tumor necrosis factor-α, ln(TNF-α) natural logarithm of TNF-α, RF rheumatoid factor, ACPA anti-citrullinated protein antibody, ESR erythrocyte sedimentation rate, IL-6 interleukin-6, MTX methotrexate, LEF leflunomide, bDMARDs biological disease-modifying antirheumatic drugs, tsDMARDs targeted synthetic disease-modifying antirheumatic drugs, OR odds ratio, CI confidence interval

Supplementary Table S8. Sensitivity analysis for the association between serum TNF-α and RA-ILD after excluding patients with bDMARDs or tsDMARDs use within the past year

**Exposure**	**Model I**<br>**OR (95%CI)**	**Model II**<br>**OR (95%CI)**	**Model III**<br>**OR (95%CI)**	**Model IV**<br>**OR (95%CI)**
**Male**				
N (Events)	167 (51)	166 (51)	167(51)	167 (51)
ln(TNF-α) (per 1-unit)	1.01 (0.79, 1.28)	1.00 (0.78, 1.29)	0.98 (0.76, 1.25)	1.03 (0.80, 1.31)
TNF-α (per 1-SD)	1.15 (0.84, 1.57)	1.17 (0.84, 1.62)	1.12 (0.81, 1.53)	1.16 (0.85, 1.60)
TNF-α Tertiles				
Low	*Ref*	*Ref*	*Ref*	*Ref*
Middle	0.88 (0.39, 1.99)	0.88 (0.38, 2.01)	0.81 (0.35, 1.89)	0.91 (0.40, 2.10)
High	0.84 (0.37, 1.92)	0.83 (0.36, 1.93)	0.75 (0.32, 1.75)	0.86 (0.37, 2.02)
P for trend	0.68	0.66	0.50	0.73
**Female**				
N (Events)	561 (85)	551 (84)	558 (85)	561(85)
ln(TNF-α) (per 1-unit)	1.43 (1.19, 1.72)	1.35 (1.11, 1.64)	1.41 (1.17, 1.70)	1.42 (1.18, 1.72)
TNF-α (per 1-SD)	1.43 (1.19, 1.72)	1.35 (1.12, 1.64)	1.42 (1.17, 1.73)	1.42 (1.18, 1.70)
TNF-α Tertiles				
Low	*Ref*	*Ref*	*Ref*	*Ref*
Middle	1.00 (0.52, 1.93)	1.00 (0.51, 1.94)	1.03 (0.53, 1.99)	1.04 (0.53, 2.02)
High	2.44 (1.35, 4.39)	2.12 (1.16, 3.87)	2.43 (1.34, 4.41)	2.50 (1.38, 4.53)
P for trend	<0.01	0.01	<0.01	<0.01

Model I was adjusted for age and disease duration in both sexes, with additional adjustment for smoking (pack-year) in males.
Model II was adjusted for Model I covariates plus RF and ACPA.
Model III was adjusted for Model I covariates plus ESR and IL-6.
Model IV was adjusted for Model I covariates plus cumulative GCs exposure, cumulative exposure duration of MTX and LEF.
Note: The number of patients included in each model using complete-case analysis, and tertile cutoffs were derived from sex-specific distributions of TNF-α values.
Abbreviations: TNF-α tumor necrosis factor α, ln(TNF-α) natural logarithm of TNF-α, RA-ILD rheumatoid arthritis associated interstitial lung disease, RF rheumatoid factor, ACPA anti-citrullinated protein antibody, ESR erythrocyte sedimentation rate, IL-6 interleukin-6, MTX methotrexate, LEF leflunomide, bDMARDs biological disease-modifying antirheumatic drugs, tsDMARDs targeted synthetic disease-modifying antirheumatic drugs, GCs glucocorticoids, OR odds ratio, CI confidence interval

Supplementary Table S9. Sensitivity analysis for TNF-α and RA-ILD after excluding pre-existing ILD (N=756)

Exposure	Model 1<br>OR (95%CI)	Model 2<br>OR (95%CI)	Model 3<br>OR (95%CI)	Model 4<br>OR (95%CI)
**Male**				
N (Events)	162 (41)	162 (41)	162 (41)	162 (41)
ln(TNF-α) (per 1-unit)	1.04 (0.80, 1.36)	1.04 (0.80, 1.36)	1.06 (0.81, 1.39)	1.01 (0.78, 1.32)
TNF-α (per 1-SD)	1.16 (0.84, 1.62)	1.16 (0.83, 1.63)	1.18 (0.85, 1.65)	1.14 (0.82, 1.59)
TNF-α Tertiles				
Low	*Ref*	*Ref*	*Ref*	*Ref*
Middle	1.07 (0.45, 2.55)	1.07 (0.45, 2.55)	1.12 (0.46, 2.71)	1.02 (0.43, 2.43)
High	0.84 (0.34, 2.08)	0.83 (0.33, 2.07)	0.89 (0.35, 2.26)	0.77 (0.30, 1.93)
P for trend	0.72	0.70	0.81	0.58
**Female**				
N (Events)	594 (74)	583 (73)	591 (74)	594 (74)
ln(TNF-α) (per 1-unit)	1.31 (1.08, 1.59)	1.26 (1.03, 1.54)	1.29 (1.06, 1.58)	1.31 (1.08, 1.60)
TNF-α (per 1-SD)	1.37 (1.13, 1.66)	1.33 (1.09, 1.63)	1.37 (1.12, 1.67)	1.38 (1.14, 1.67)
TNF-α Tertiles				
Low	*Ref*	*Ref*	*Ref*	*Ref*
Middle	0.85 (0.43, 1.69)	0.85 (0.43, 1.70)	0.86 (0.43, 1.72)	0.88 (0.44, 1.75)
High	1.92 (1.08, 3.51)	1.73 (1.02, 3.22)	1.89 (1.04, 3.48)	1.96 (1.06, 3.61)
P for trend	0.02	0.04	0.03	0.02

Model I was adjusted for age and disease duration in both sexes, with additional adjustment for smoking (pack-year) in males.
Model II was adjusted for Model I covariates plus RF and ACPA.
Model III was adjusted for Model I covariates plus ESR and IL-6.
Model IV was adjusted for Model I covariates plus cumulative exposure duration of LEF and other bDMARDs/tsDMARDs in males, and further adjusted for cumulative GCs exposure, MTX, LEF, and other bDMARDs/tsDMARDs exposure duration in females.
Note: The number of patients included in each model using complete-case analysis, and tertile cutoffs were derived from sex-specific distributions of TNF-α values.
Abbreviations: TNF-α tumor necrosis factor α, ln(TNF-α) natural logarithm of TNF-α, RA-ILD rheumatoid arthritis associated interstitial lung disease, RF rheumatoid factor, ACPA anti-citrullinated protein antibody, ESR erythrocyte sedimentation rate, IL-6 interleukin-6, GCs glucocorticoids, MTX methotrexate, LEF leflunomide, bDMARDs biological disease-modifying antirheumatic drugs, tsDMARDs targeted synthetic disease-modifying antirheumatic drugs, OR odds ratio, CI confidence interval

Supplementary Table S10. Sensitivity analysis for the association between TNF-α and RA-ILD using pooled tertiles

Exposure	Model I<br>OR (95%CI)	Model II<br>OR (95%CI)	Model III<br>OR (95%CI)	Model IV<br>OR (95%CI)
**Male**				
N (Events)	176 (54)	175 (54)	176 (54)	176 (54)
TNF-α Tertiles				
Low	*Ref*	*Ref*	*Ref*	*Ref*
Middle	1.23 (0.54, 2.81)	1.27 (0.55, 2.90)	1.23 (0.54, 2.81)	1.27 (0.55, 2.90)
High	0.98 (0.44, 2.15)	0.89 (0.40, 2.00)	0.98 (0.44, 2.15)	0.89 (0.40, 2.00)
P for trend	0.94	0.79	0.94	0.79
**Female**				
N (Events)	614 (95)	603 (94)	611 (95)	614 (95)
TNF-α Tertiles				
Low	*Ref*	*Ref*	*Ref*	*Ref*
Middle	1.19 (0.65, 2.20)	1.21 (0.66, 2.24)	1.21 (0.65, 2.23)	1.25 (0.68, 2.33)
High	2.20 (1.24, 3.91)	1.90 (1.05, 3.43)	2.17 (1.21, 3.87)	2.33 (1.30, 4.16)
P for trend	< 0.01	< 0.01	< 0.01	< 0.01

Model I was adjusted for age and disease duration in both sexes, with additional adjustment for smoking (pack-year) in males.
Model II was adjusted for Model I covariates plus RF and ACPA.
Model III was adjusted for Model I covariates plus ESR and IL-6.
Model IV was adjusted for Model I covariates plus cumulative exposure duration of LEF and other bDMARDs/tsDMARDs in males, and cumulative GCs exposure, MTX, LEF, and other bDMARDs/tsDMARDs cumulative exposure duration in females.
Note: The number of patients included in each model using complete-case analysis, and tertile cutoffs were calculated based on the entire cohort distribution.
Abbreviations: TNF-α tumor necrosis factor α, RA-ILD rheumatoid arthritis associated interstitial lung disease,

RF rheumatoid factor, ACPA anti-citrullinated protein antibody, ESR erythrocyte sedimentation rate, IL-6 interleukin-6, MTX methotrexate, LEF leflunomide, bDMARDs biological disease-modifying antirheumatic drugs, tsDMARDs targeted synthetic disease-modifying antirheumatic drugs, GCs glucocorticoids, OR odds ratio, CI confidence interval
