# Supplementary material for: Association between serum tumor necrosis factor-alpha levels and rheumatoid arthritis-associated interstitial lung disease: a cross-sectional study
Source: Front Immunol. 2026 Aug 26;17:1881556. doi: 10.3389/fimmu.2026.1881556 (PMC13553761; doi:10.3389/fimmu.2026.1881556)
Supplement: Supplementary file 2 [file DataSheet2.pdf]

Supplementary_Material_R code for the main statistical analyses

Supplementary Code S1. R code for multivariable logistic regression and generalized additive models (GAM) for the association between TNF-α and RA-ILD (Table 3)

```
#This code demonstrates the analytical workflow for Model 1 and Model 2 using the female dataset.
#Model 3 and Model 4 differ only in the adjustment variables (Model 3: +ESR, IL-6; Model 4: +medication variables),
#While the model structure and code logic remain identical.
#The same analytical approach applies to the male data, with the only change being the replacement of the dataset with male.Rdata.

#******************** Regarding ALL Following R Code      ********************
#*** COPYRIGHT (c) 2010, 2021 X&Y Solutions, ALL RIGHT RESERVED ***********
#****************** www.EmpowerStats.com *********************************
#**********************************************************************
Sys.setlocale(category = 'LC_ALL', locale = 'English_United States.1252');
.libPaths(file.path(R.home(),'library'));
library(doBy);
options(timeout=600);
library(plotrix);
library(stringi);
library(stringr);
library(survival);
library(rms);
library(nnet);
library(car);
library(mgcv);
pdfwd<-6; pdfht<-6;
load('D:/EmpowerStats/Analysis/female/female.Rdata');
if (length(which(ls()=='EmpowerStatsR'))==0) EmpowerStatsR<-get(ls()[1]);
names(EmpowerStatsR)<-toupper(names(EmpowerStatsR));
originalVNAME<-names(EmpowerStatsR);
ofname<-'female_1_tbl';
rankvar <- function(var, num) {
   qprobs <- 1/num
   if (num>2) {for (i in (2:(num-1))) {qprobs <- c(qprobs, 1/num * i) }}
   outvar <- rep(0, times=length(var))
   outvar[is.na(var)] <- NA
   cutpoints <- quantile(var,probs=qprobs, na.rm=TRUE)
   for (k in (1:length(cutpoints))) {outvar[var>=cutpoints[k]] <- k}
   tmp<-c(min(var,na.rm=TRUE),cutpoints,max(var,na.rm=TRUE))
   names(tmp)<-c("Min",names(cutpoints),"Max")
```

```
    print(tmp)
    return(outvar)
}
g2idx<-function(x) {
  g<-levels(factor(x));    y<-rep(NA,length(x))
  for (i in 1:length(g)) y[x==g[i]]<-i;
  y[is.na(y)]<-length(g)+1;
  return(y);
}
zscorevname<-function(xvname, gvname, mu, std, ref, stratum=c()) {
  tmpd<-EmpowerStatsR[,xvname]; tmpg_<-rep(1,length(tmpd));
  ifelse(gvname>"", tmpd<-cbind(tmpd,EmpowerStatsR[,gvname]), tmpd<-cbind(tmpd,tmpg_));
  ng<-length(levels(factor(tmpd[,2])))
  if (ref>"") {
      tmpd0<-EmpowerStatsR[eval(parse(text=ref)),xvname]; tmpg0_<-rep(1,length(tmpd0));
      ifelse(gvname>"",          tmpd0<-cbind(tmpd0,EmpowerStatsR[eval(parse(text=ref)),gvname]),
tmpd0<-cbind(tmpd0,tmpg0_));
  } else { tmpd0<-tmpd; }
  ng0<-length(levels(factor(tmpd0[,2])))
  if (ng!=ng0) tmpd0<-tmpd
  tmpd[,2]<-g2idx(tmpd[,2]); tmpd0[,2]<-g2idx(tmpd0[,2])
  nmu<-length(mu)
  if (nmu==0 | nmu!=length(std) | !is.numeric(mu) | !is.numeric(std) | nmu!=ng) mu<-c()
  if (length(stratum)>0) {
      if (length(mu) != length(stratum)) mu <- c()
  }
  if (length(mu)==0) {
      mu<-c(tapply(tmpd0[,1],tmpd0[,2], mean, na.rm=TRUE))
      std<-c(tapply(tmpd0[,1],tmpd0[,2], sd, na.rm=TRUE))
  } else {
      if (length(stratum)>0) {
            ord <- match(stratum, as.numeric(levels(factor(tmpd0[,2]))))
            mu <- mu[ord]
            std <- std[ord]
      }
  }
  print("Mean:"); print(mu); print("SD:"); print(std);
  y<-(tmpd[,1]-mu[tmpd[,2]])/std[tmpd[,2]];
  return(y);
}
attach(EmpowerStatsR);
sink(paste(ofname,'_datastep.lst',sep=''));
print('Creating new variable: ZV.FML');
ZV.FML<-X25+1;
```

```
summary(ZV.FML);
EmpowerStatsR<-cbind(EmpowerStatsR,ZV.FML);
print('Creating new variable: ZV.FML.LOG');
ZV.FML.LOG<- ifelse(ZV.FML>0, log(ZV.FML), NA);
summary(ZV.FML.LOG);
EmpowerStatsR<-cbind(EmpowerStatsR,ZV.FML.LOG);
print('Creating new variable: X25.Z');
X25.Z<- zscorevname("X25","",c(),c(),"",c());
summary(X25.Z);
EmpowerStatsR<-cbind(EmpowerStatsR,X25.Z);
print('Creating new variable: X25.T3');
X25.T3<- rankvar(X25,3);
summary(X25.T3);
EmpowerStatsR<-cbind(EmpowerStatsR,X25.T3);
print('Creating new variable: X25.T3.CONT');
X25.T3.CONT<- X25.T3;
summary(X25.T3.CONT);
EmpowerStatsR<-cbind(EmpowerStatsR,X25.T3.CONT);
rm(ZV.FML,ZV.FML.LOG,X25.Z,X25.T3,X25.T3.CONT);
sink();
vname<-c(NA,'X3','X12','X12.0','X12.1','X18','X23','X25','X26','ZV.FML','ZV.FML.LOG','X25.Z','X25.T
3','X25.T3.0','X25.T3.1','X25.T3.2','X25.T3.CONT')[-1];
vlabel<-c(NA,'Age','RA-ILD','    No','    Yes','Duration','RF','TNF-a','CCP','ZV.FML','ln(TNF α )','TNF-a
Z-score','TNF-a tertile','    Low','    Middle','    High','TNF-a tertile continuous')[-1];
varused4this <-
c('X3','X12','X18','X23','X25','X26','ZV.FML','ZV.FML.LOG','X25.Z','X25.T3','X25.T3.CONT');
pkgs<-c('gdata','geepack','mgcv');
for (g in pkgs) {
if (!(g %in% rownames(installed.packages())))
install.packages(g,repos='https://cloud.r-project.org');
}
library(gdata);
library(geepack);
library(mgcv);
WD <- EmpowerStatsR; rm(EmpowerStatsR); gc();
title<-'Mutiple regressions';
wd.subset='';
weights.var<- NA;
yvname<-c('X12');
ydist<-c('binomial');
ylink<-c('logit');
ylv<-c(2);
avname<-c('X3','X18');
saf<-c(0,0);
```

```
alv<-c(0,0);
svname<-c('X3','X18','X23','X26');
sdf<-c(0,0,0,0);
slv<-c(0,0,0,0);
prn<-1;
xvname<-c('ZV.FML.LOG','X25.Z','X25.T3','X25.T3.CONT');
sxf<-c(0,0,0,0);
xlv<-c(0,0,3,0);
par1<-1;
chk<- 0;
cox<- 0;
timevar<- NA;
vname.start<- NA;
subjvname<- NA;
gee.TYPE<-c('independence', 'unstructured', 'exchangeable', 'AR1')[0];
bvar<- NA;
colvname<- NA;
par3<-1;
dec<-2;
##R package## gdata geepack mgcv ##R package##;
pvformat<-function(p,dec) {
  pp <- sprintf(paste("%.",dec,"f",sep=""),as.numeric(p))
  if (is.matrix(p)) {pp<-matrix(pp, nrow=nrow(p));
colnames(pp)<-colnames(p);rownames(pp)<-rownames(p);}
  lw <- paste("<",substr("0.00000000000",1,dec+1),"1",sep="");
  pp[as.numeric(p)<(1/10^dec)]<-lw
  return(pp)
}
numfmt<-function(p,dec) {
  if (is.list(p)) p<-as.matrix(p)
  pp <- sprintf(paste("%.",dec,"f",sep=""),as.numeric(p))
  if (is.matrix(p)) {pp<-matrix(pp,
nrow=nrow(p));colnames(pp)<-colnames(p);rownames(pp)<-rownames(p);}
  pp[as.numeric(p)>10000000]<- "inf."
  pp[is.na(p) | gsub(" ","",p)==""]<- ""
  pp[p=="-Inf"]<-"-Inf"
  pp[p=="Inf"]<-"Inf"
  return(pp)
}
varstats<-function(var,vlvl,dec) {
  if (length(vlvl)==1 & vlvl[1]==0) {

return(paste(numfmt(mean(var,na.rm=TRUE),dec),numfmt(sd(var,na.rm=TRUE),dec),sep="+"))
  } else {
```

```
    a<-table(var)
    b<-matrix(paste(a, " (", numfmt(a/sum(a)*100,dec), "%)",sep=""),ncol=1)
    return(c(" ",b[match(vlvl,names(a))]))
  }
}
mat2htmltable<-function(mat) {
  t1<- apply(mat,1,function(z) paste(z,collapse="</td><td>"))
  t2<- paste("<tr><td>",t1,"</td></tr>")
  return(paste(t2,collapse=" "))
}
setgam<-function(fml,yi) {
  if (ydist[yi]=="") ydist[yi]<-"gaussian"
  if (ydist[yi]=="exact") ydist[yi]<-"binomial"
  if (ydist[yi]=="breslow") ydist[yi]<-"binomial"
  if (ydist[yi]=="gaussian") mdl<-try(gam(formula(fml),weights=wdtmp$weights,data=wdtmp, family=gaussian(link="identity")))
  if (ydist[yi]=="binomial") mdl<-try(gam(formula(fml),weights=wdtmp$weights,data=wdtmp, family=binomial(link="logit")))
  if (ydist[yi]=="poisson") mdl<-try(gam(formula(fml),weights=wdtmp$weights,data=wdtmp, family=poisson(link="log")))
  if (ydist[yi]=="gamma") mdl<-try(gam(formula(fml),weights=wdtmp$weights,data=wdtmp, family=Gamma(link="inverse")))
  if (ydist[yi]=="negbin") mdl<-try(gam(formula(fml),weights=wdtmp$weights,data=wdtmp, family=negbin(c(1,10), link="log")))
  return(mdl)
}
setgee<-function(fml,yi) {
  if (ydist[yi]=="") ydist[yi]<-"gaussian"
  if (ydist[yi]=="exact") ydist[yi]<-"binomial"
  if (ydist[yi]=="breslow") ydist[yi]<-"binomial"
  if (ydist[yi]=="gaussian") md<-try(geeglm(formula(fml),id=wdtmp[,subjvname],corstr=gee.TYPE,family="gaussian",weights=wdtmp$weights,data=wdtmp))
  if (ydist[yi]=="binomial") md<-try(geeglm(formula(fml),id=wdtmp[,subjvname],corstr=gee.TYPE,family="binomial",weights=wdtmp$weights,data=wdtmp))
  if (ydist[yi]=="poisson") md<-try(geeglm(formula(fml),id=wdtmp[,subjvname],corstr=gee.TYPE,family="poisson",weights=wdtmp$weights,data=wdtmp))
  if (ydist[yi]=="gamma") md<-try(geeglm(formula(fml),id=wdtmp[,subjvname],corstr=gee.TYPE,family="Gamma",weights=wdtmp$weights,data=wdtmp))
  if (ydist[yi]=="negbin") md<-try(geeglm.nb(formula(fml),id=wdtmp[,subjvname],corstr=gee.TYPE,weights=wdtmp$weigh
```

```
ts,data=wdtmp))
   return(md)
}
setglm<-function(fml,yi) {
   if (ydist[yi]=="") ydist[yi]<-"gaussian"
   if (ydist[yi]=="exact") ydist[yi]<-"binomial"
   if (ydist[yi]=="breslow") ydist[yi]<-"binomial"
   if (ydist[yi]=="gaussian")
md<-try(glm(formula(fml),family="gaussian",weights=wdtmp$weights,data=wdtmp))
   if (ydist[yi]=="binomial")
md<-try(glm(formula(fml),family="binomial",weights=wdtmp$weights,data=wdtmp))
   if (ydist[yi]=="poisson")
md<-try(glm(formula(fml),family="poisson",weights=wdtmp$weights,data=wdtmp))
   if (ydist[yi]=="gamma")
md<-try(glm(formula(fml),family="Gamma",weights=wdtmp$weights,data=wdtmp))
   if (ydist[yi]=="negbin") md<-try(glm.nb(formula(fml),weights=wdtmp$weights,data=wdtmp))
   return(md)
}
mdl2oo<-function(mdl, xxname, opt) {
   if (is.na(mdl[[1]][1])) return(list(rep("",times=length(xxname)),""))
   if (substr(mdl[[1]][1],1,5)=="Error") return(list(rep("",times=length(xxname)),""))
   gs<-summary(mdl); print(mdl$formula); print(gs)
   if (opt=="gam") {gsparm <- gs$p.table;tmpn<-gs$n;
   } else {gsparm <- gs$coefficients;tmpn <- sum(gs$df[c(1,2)]);}
   gsp<-gsparm[match(xxname,rownames(gsparm)),]
   if (length(xxname)==1) {beta<-gsp[1]; se<-gsp[2]; pv<-gsp[4];
   } else {beta<-gsp[,1]; se<-gsp[,2]; pv<-gsp[,4]; }
   ci1<- beta-1.96*se; ci2<- beta+1.96*se
   pvx<-substr(rep("****",length(pv)),1,(pv<=0.05)+(pv<=0.01)+(pv<=0.001))
   if (colprn==3) {pvv<-pvx;} else {pvv<-pvformat(pv,dec+2);}
   if ((colprn!=2) & (gs$family[[2]]=="log" | gs$family[[2]]=="logit")) {
      o1<-paste(numfmt(exp(beta),dec)," (",numfmt(exp(ci1),dec),",
",numfmt(exp(ci2),dec),")",sep="")
   } else {
      if (colprn<3) {o1<-paste(numfmt(beta,dec), " (",numfmt(ci1,dec),",
",numfmt(ci2,dec),")",sep="")
      } else {o1<-paste(numfmt(beta,dec), "+",numfmt(se,dec),sep="");}
   }
   o1<-paste(o1,pvv);    o1[is.na(beta)]<-NA
   if (length(xxname)>1) {
      if (gs$family[[2]]=="log" | gs$family[[2]]=="logit") {
          o1[is.na(o1) & substr(xxname,1,7)=="factor("]<-"1.0"
      } else {o1[is.na(o1) & substr(xxname,1,7)=="factor("]<-"0";}
      o1[is.na(o1)]<-"";
```

```
    }
    return(list(o1,tmpn))
}
recodevar <- function (var,oldcode,newcode) {
    tmp.v <- var
    nc.tmp <- length(oldcode)
    for (i in (1:nc.tmp)) {tmp.v[(var==oldcode[i])]=newcode[i]}
    if (is.factor(tmp.v)) {tmp.v1<-as.numeric(as.character(tmp.v))} else
{tmp.v1<-as.numeric(tmp.v)}
    rm(tmp.v);    return(tmp.v1)
}
rankvar <- function(var, num) {
    qprobs <- 1/num
    if (num>2) {for (i in (2:(num-1))) {qprobs <- c(qprobs, 1/num * i) } }
    outvar <- rep(0, times=length(var))
    outvar[is.na(var)] <- NA
    cutpoints <- quantile(var,probs=qprobs, na.rm=TRUE)
    for (k in (1:length(cutpoints))) { outvar[var>=cutpoints[k]] <- k; }
    return(outvar)
}
removeNA<-function(i,j,m,wdf) {
  vvv<-c(yvname[i],adjvv[[m]],subjvname,colvname,bvar,vname.start,timevar);
  if (j<=nx) {vvv<-c(vvv,xvname[j]);} else {vvv<-c(vvv,xvname);}
  vvv<-vvv[!is.na(vvv)]; vvv<-vvv[vvv>" "]
  tmp<-is.na(wdf[,vvv]);
  return(wdf[apply(tmp,1,sum)==0,])
}
if (!is.na(weights.var)) {weights<-WD[,weights.var];} else {weights<-1;}
WD<-cbind(WD,weights);
vlabelN<-(substr(vlabel,1,1)==" ");
vlabelZ<-vlabel[vlabelN];vlabelV<-vlabel[!vlabelN]
vnameV<-vname[!vlabelN];vnameZ<-vname[vlabelN]
w<-c("<!DOCTYPE html><html lang='zh'><head><meta charset='utf-8'></head><body>")
w<-c(w,paste("<h2>", title, "</h2>"))
if (length(avname)>0) {
  if (sum((saf=="s" | saf=="S") & alv>0)>0) w<-c(w,"</br>Spline smoothing only applies for
continuous variables")
  if (!is.na(subjvname) & (sum((saf=="s" | saf=="S") & alv==0)>0)) w<-c(w,"</br>Generalized
estimate equation could not be used with spline smoothing terms")
}
if (length(svname)>0) {
  if (sum((sdf=="s" | sdf=="S") & slv>0)>0) w<-c(w,"</br>Spline smoothing only applies for
continuous variables")
  if (!is.na(subjvname) & (sum((sdf=="s" | sdf=="S") & slv==0)>0)) w<-c(w,"</br>Generalized
```

```
estimate equation could not be used with spline smoothing terms")
}
allvname<-c(yvname,xvname,colvname,bvar,avname,svname,subjvname,vname.start,timevar,"w
eights");
allvname<-allvname[!is.na(allvname)]
WD<-WD[,allvname];
if (!is.na(subjvname)) WD<-WD[order(WD[,subjvname]),]
sxf<-as.numeric(sxf);sxf[is.na(sxf)]<-0;
if (sum(sxf>1 & xlv>0)>0) w<-c(w,"Categorizing only applies to continuous variables");
if (sum(sxf>1 & xlv==0)>0) {
        t.xname<-NA;t.xlv<-NA; nx<-length(xvname)
        for (i in 1:nx) {
           if (sxf[i]>1 & xlv[i]==0) {
                tmp.Xi<- rankvar(WD[,xvname[i]],sxf[i])
                tmp.newcode <- tapply(WD[,xvname[i]],tmp.Xi,function(z) median(z,na.rm=TRUE))
                tmp.low <- tapply(WD[,xvname[i]],tmp.Xi,function(z) min(z,na.rm=TRUE))
                tmp.upp <- tapply(WD[,xvname[i]],tmp.Xi,function(z) max(z,na.rm=TRUE))
                tmp.Xi2<- recodevar(tmp.Xi,(1:sxf[i])-1,tmp.newcode)
                tmp.Xi<-cbind(tmp.Xi,tmp.Xi2)
                tmp.NM<-paste(xvname[i],c("grp","grp.cont"),sep=".")
                colnames(tmp.Xi)<-tmp.NM
                WD<-cbind(WD,tmp.Xi)
                t.xname<-c(t.xname,tmp.NM)
                t.xlv<-c(t.xlv,sxf[i],0)
                vnameV<-c(vnameV,tmp.NM)
                vlabelV<-c(vlabelV,paste(vlabelV[vnameV==xvname[i]],c("group","group trend")))
                vnameZ<-c(vnameZ,paste(tmp.NM[1],(1:sxf[i])-1,sep="."))
                vlabelZ<-c(vlabelZ,paste(tmp.low,"-",tmp.upp))
           } else {
                t.xname<-c(t.xname,xvname[i]); t.xlv<-c(t.xlv,xlv[i])
           }
        }
        xvname<-t.xname[-1]; xlv<-t.xlv[-1];
}
if (!is.na(subjvname)) {
  if (length(avname)>0) saf<-rep(0,length(saf));
  if (length(svname)>0) sdf<-rep(0,length(sdf));
  WD<-WD[order(WD[,subjvname]),];
}
ny=length(yvname); nx=length(xvname);
xb<-vlabelV[match(xvname,vnameV)]; xb[is.na(xb)]<-xvname[is.na(xb)]
yb<-vlabelV[match(yvname,vnameV)]; yb[is.na(yb)]<-yvname[is.na(yb)]
xvname_ <- xvname
xvname_[xlv>0]<-paste("factor(",xvname[xlv>0],")",sep="")
```

```
xxname_<-list(NA); xxlbl_<-list(NA); xxlvl_<-list(NA)
for (j in (1:nx)) {
  if (xlv[j]==0) {
    xxname_[[j+1]]<-xvname[j];xxlbl_[[j+1]]<-xb[j];xxlvl_[[j+1]]<-0
  } else {
    xxlvl_[[j+1]]<-levels(factor(WD[,xvname[j]]))
    tmp<-paste(xvname[j],".",xxlvl_[[j+1]],sep="")
    xxlbl_[[j+1]]<-c(xb[j],vlabelZ[match(tmp,vnameZ)])
    xxlbl_[[j+1]]<-paste(c("",rep("??",length(xxlbl_[[j+1]])-1)),xxlbl_[[j+1]])
    xxname_[[j+1]]<-c(xvname[j],paste("factor(",xvname[j],")",xxlvl_[[j+1]],sep=""))
  }
}
xxname_<-xxname_[-1]; xxlbl_<-xxlbl_[-1]; xxlvl_<-xxlvl_[-1];
if (nx==1) par1<-1;
if (nx!=length(svname) & par1==4) par1<-1;
if (is.na(par1) | par1<1) par1<-1;
if (par1>1 & par1 < 4) {
  tmp1<-xxname_[[1]]; tmp2<-xxlbl_[[1]]
  for (j in 2:nx) {tmp1<-c(tmp1,xxname_[[j]]); tmp2<-c(tmp2,xxlbl_[[j]]);}
  xxname_[[nx+1]]<-tmp1; xxlbl_[[nx+1]]<-tmp2;
  xvname_<-c(xvname_,paste(xvname_,collapse="+"))
}
contx<-(sum(xlv>0)==0)
if (par1==3 & !is.na(bvar)) {w<-c(w,"</br>Column stratified variable was ignored"); bvar<-NA;
bvname<-NA;}

fmlm<-" "; fmlb<-"Non-adjusted"; tmp<-""; adjvv<-list(NA); adjvb<-"None";
fmlm4<-list(rep("",nx))
fmlp<-ifelse(!is.na(subjvname), "gee", "glm");
na=0; avb=""; smoothav<-0; nadjm<-0
if (length(avname)>0) {
  na<-length(avname)
  avb<-vlabelV[match(avname,vnameV)];
  avname_ <- avname
  smoothavi<-((saf=="s" | saf=="S") & alv==0)
  smoothav<-sum(smoothavi)
  smoothavname<-avname[smoothavi]
  avname_[smoothavi]<-paste("s(",avname[smoothavi],")",sep="")
  avb1<-avb
  avb1[smoothavi]<-paste(avb[smoothavi],"(Smooth)",sep="")
  avname_[alv>0]<-paste("factor(",avname[alv>0],")",sep="")
  fmlm<-c(fmlm,paste("+",paste(avname_,collapse="+")))
  fmlb<-c(fmlb,"Adjust")
  nadjm<-nadjm+1; tmp<-c(tmp,"I"); adjvv[[nadjm+1]]<-avname;
```

```
    adjvb<-c(adjvb, paste(avb1, collapse="; "))
    fmlp<-c(fmlp,ifelse(!is.na(subjvname), "gee", ifelse(smoothav>0, "gam", "glm")))
    fmlm4<-c(fmlm4, list(rep("",nx)))
}
ns=0; svb=""; smoothsv<-0

if (length(svname)>0) {
    svb<-vlabelV[match(svname,vnameV)];
    svname_ <- svname
    smoothsvi<-((sdf=="s" | sdf=="S") & slv==0)
    smoothsv<-sum(smoothsvi)
    smoothsvname<-svname[smoothsvi]
    svname_[smoothsvi]<-paste("s(",svname[smoothsvi],")",sep="")
    svb1<-svb
    svb1[smoothsvi]<-paste(svb[smoothsvi],"(Smooth)",sep="")
    svname_[slv>0]<-paste("factor(",svname[slv>0],")",sep="")
    if (par1==4) {
        fmlm<-c(fmlm, paste(fmlm[length(fmlm)],"+"));
        fmlm4<-c(fmlm4, list(svname_));
    } else {
        fmlm<-c(fmlm,paste("+",paste(svname_,collapse="+")));
        fmlm4<-c(fmlm4, list(rep("",nx)));
    }
    fmlb<-c(fmlb,"Adjust")
    nadjm<-nadjm+1; tmp<-c(tmp,"II"); adjvv[[nadjm+1]]<-svname
    adjvb2 <- paste(svb1, collapse="; ")
    if (par1 == 4) {
         if (avb>"") {adjvb2<-paste(avb1, "AND one in (", adjvb2,") respectively for each exposure
X");
         } else {          adjvb2<-paste("ONE of (", adjvb2,") respectively for each exposure X");}
    }
    adjvb<-c(adjvb, adjvb2)
    fmlp<-c(fmlp,ifelse(!is.na(subjvname), "gee", ifelse(smoothsv>0, "gam", "glm")))
}
if (chk==0 & length(fmlm)>1) {
    fmlm<-fmlm[-1];      fmlb<-fmlb[-1];      tmp<-tmp[-1];      adjvv<-adjvv[-1];      adjvb<-adjvb[-1];
fmlp<-fmlp[-1]; fmlm4<-fmlm4[-1];
}
if (nadjm>1) fmlb<-paste(fmlb,tmp)
nmdl<-length(fmlm)
if (is.na(bvar) & !is.na(colvname) & nmdl==1 & par1!=3) {if ((ny==1) | (nx==1 & contx))
{bvar<-colvname; colvname<-NA;}}
if (is.na(colvname)) {
    nclv<-1; clvb<-"Total"; clvb_<-"Total"
```

```
} else {
   clv<-levels(factor(WD[,colvname])); nclv<-length(clv)+1
   clvb_<-vlabelZ[match(paste(colvname,".",clv,sep=""),vnameZ)];
clvb_[is.na(clvb_)]<-clv[is.na(clvb_)];
   clvb<-c(paste(vlabelV[vnameV==colvname],clvb_,sep="="),"Total");
   clvb_<-c(clvb_,"Total")
   WD<-WD[!is.na(WD[,colvname]),]
}
if (is.na(bvar)) {
   blvb<-"Total"; blvb_<-"Total"
} else {
   blv<-levels(factor(WD[,bvar])); nblv<-length(blv)+1
   blvb_<-vlabelZ[match(paste(bvar,".",blv,sep=""),vnameZ)];
blvb_[is.na(blvb_)]<-blv[is.na(blvb_)];
   blvb<-c(paste(vlabelV[vnameV==bvar],blvb_,sep="="),"Total");
   blvb_<-c(blvb_,"Total")
   WD<-WD[!is.na(WD[,bvar]),]
}
aa<-c(1,2,3,4)
for (i in 1:4) {
         for (j in c(1:4)[-i]) {
               for (k in c(1:4)[-c(i,j)]) aa<-rbind(aa,c(i,j,k,c(1:4)[-c(i,j,k)]))
         }
}
colprn<-prn;
if (is.na(par3) | par3<1) par3<-1
rord<-aa[par3,]
if (!is.na(bvar)) {prn<-"S";
} else {
   if (par3>1) {
      rordc<-ifelse(rord[4]==1,3,4)
      prn<-c("G","Y","M","X")[rord[rordc]]
      if (prn=="X") {
         if (!contx & nx>1) prn<-ifelse(nmdl>1, "M", ifelse(ny>nx & contx, "X", "Y"))
         if (!contx & nx==1) prn<-"CX"
         if          (par1==2)          {tmp<-ifelse(rordc==4,ifelse(rord[3]==1,2,3),2);
prn<-c("G","Y","M","X")[rord[tmp]];}
      }
   } else {
      prn<-ifelse(nmdl>1, "M", ifelse(ny>nx & contx, "X", "Y"))
      if (par1==2) prn<-ifelse(nmdl>1, "M", "Y")
   }
   if (par1==3) prn<-"UM"
}
```

```
sink(paste(ofname,".lst",sep=""))
if (par1==2) {xbgn<-nx+1; xend<-nx+1;} else {xbgn<-1; xend<-nx;}
if (prn=="Y") {
   tt<-c(0,0,0,0,"Exposure",yb); nn<-c(0,0,0,0,yb);
   for (k in (1:nclv)) {
      wdtmp0<-WD;
      if (!is.na(colvname)) {
         if (k<nclv) wdtmp0<-WD[WD[,colvname]==clv[k],];
         print(paste("Stratified by",colvname, ":", clvb[k]))
      }
      for (m in 1:nmdl) {
         for (j in (xbgn:xend)) {
            colj<-cbind(k,0,m,j,xxlbl_[[j]])
            nnj <-c(k,0,m,j)
            for (i in (1:ny)) {
                  if   (par1==4)   {fml<-paste(yvname[i],"~",xvname_[j],fmlm[m],fmlm4[[m]][j]);}   else
{fml<-paste(yvname[i],"~",xvname_[j],fmlm[m]);}
               wdtmp<-removeNA(i,j,m,wdtmp0)
               if (!is.na(colvname)) {if (k==nclv) fml<-paste(fml,"+factor(",colvname,")",sep="");}
               if (fmlp[m]=="gam") tmp.mdl<-setgam(fml,i)
               if (fmlp[m]=="gee") tmp.mdl<-setgee(fml,i)
               if (fmlp[m]=="glm") tmp.mdl<-setglm(fml,i)
               tmpooi<-mdl2oo(tmp.mdl,xxname_[[j]],fmlp[m])
               colj<-cbind(colj,tmpooi[[1]]); nnj<-c(nnj,tmpooi[[2]])
            }
            tt<-rbind(tt,colj); nn<-rbind(nn,nnj)
         }
      }
   }
}
if (prn=="S") {
   tt<-c(0,0,0,0,"Exposure",blvb); nn<-c(0,0,0,0,blvb);
   for (k in (1:nclv)) {
      wdtmp0<-WD;
      if (!is.na(colvname)) {
         if (k<nclv) wdtmp0<-WD[WD[,colvname]==clv[k],];
         print(paste("Stratified by",colvname, ":", clvb[k]))
      }
      for (i in (1:ny)) {
         for (m in 1:nmdl) {
            for (j in (xbgn:xend)) {
               colj<-cbind(k,i,m,j,xxlbl_[[j]]);
               nnj <- c(k,i,m,j)
               for (b in (1:nblv)) {
```

```
                    print(paste("Stratified by",bvar, ":", blvb[b]))
                     if (par1==4) {fml<-paste(yvname[i],"~",xvname_[j],fmlm[m],fmlm4[[m]][j]);} else
{fml<-paste(yvname[i],"~",xvname_[j],fmlm[m]);}
                    if (b<nblv) {wdtmp1<-wdtmp0[wdtmp0[,bvar]==blv[b],];} else {wdtmp1<-wdtmp0;
fml<-paste(fml,"+factor(",bvar,")",sep="");}
                    wdtmp<-removeNA(i,j,m,wdtmp1)
                    if (!is.na(colvname)) {if (k==nclv) fml<-paste(fml,"+factor(",colvname,")",sep="");}
                    if (fmlp[m]=="gam") tmp.mdl<-setgam(fml,i)
                    if (fmlp[m]=="gee") tmp.mdl<-setgee(fml,i)
                    if (fmlp[m]=="glm") tmp.mdl<-setglm(fml,i)
                    tmpooi<-mdl2oo(tmp.mdl,xxname_[[j]],fmlp[m])
                    colj<-cbind(colj,tmpooi[[1]]); nnj<-c(nnj,tmpooi[[2]])
                }
                tt<-rbind(tt,colj); nn<-rbind(nn,nnj)
            }
        }
    }
  }
}
if (prn=="M") {
  tt<-c(0,0,0,0,"Exposure",fmlb);    nn<-c(0,0,0,0,fmlb)
  for (k in (1:nclv)) {
    wdtmp0<-WD;
    if (!is.na(colvname)) {
      if (k<nclv) wdtmp0<-WD[WD[,colvname]==clv[k],];
      print(paste("Stratified by",colvname, ":", clvb[k]))
    }
    for (i in 1:ny) {
      for (j in xbgn:xend) {
        colj<-cbind(k,i,0,j,xxlbl_[[j]]); nnj<-c(k,i,0,j)
        for (m in 1:nmdl) {
          if   (par1==4)   {fml<-paste(yvname[i],"~",xvname_[j],fmlm[m],fmlm4[[m]][j]);}   else
{fml<-paste(yvname[i],"~",xvname_[j],fmlm[m]);}
          wdtmp<-removeNA(i,j,m,wdtmp0)
          if (!is.na(colvname)) {if (k==nclv) fml<-paste(fml,"+factor(",colvname,")",sep="");}
          if (fmlp[m]=="gam") tmp.mdl<-setgam(fml,i)
          if (fmlp[m]=="gee") tmp.mdl<-setgee(fml,i)
          if (fmlp[m]=="glm") tmp.mdl<-setglm(fml,i)
          tmpooi<-mdl2oo(tmp.mdl,xxname_[[j]],fmlp[m])
          colj<-cbind(colj,tmpooi[[1]]); nnj<-c(nnj,tmpooi[[2]])
        }
        tt<-rbind(tt,colj); nn<-rbind(nn,nnj)
      }
    }
```

```
    }
}
if (prn=="X") {
    tt<-c(0,0,0,0,"Outcome",xb); nn<-c(0,0,0,0,xb);
    for (k in (1:nclv)) {
        wdtmp0<-WD;
        if (!is.na(colvname)) {
            if (k<nclv) wdtmp0<-WD[WD[,colvname]==clv[k],];
            print(paste("Stratified by",colvname, ":", clvb[k]))
        }
        for (m in 1:nmdl) {
            for (i in (1:ny)) {
                colj<-cbind(k,i,m,0,yb[i])
                nnj <-c(k,i,m,0)
                for (j in (1:nx)) {
                    if   (par1==4)   {fml<-paste(yvname[i],"~",xvname_[j],fmlm[m],fmlm4[[m]][j]);}   else
{fml<-paste(yvname[i],"~",xvname_[j],fmlm[m]);}
                    wdtmp<-removeNA(i,j,m,wdtmp0)
                    if (!is.na(colvname)) {if (k==nclv) fml<-paste(fml,"+factor(",colvname,")",sep="");}
                    if (fmlp[m]=="gam") tmp.mdl<-setgam(fml,i)
                    if (fmlp[m]=="gee") tmp.mdl<-setgee(fml,i)
                    if (fmlp[m]=="glm") tmp.mdl<-setglm(fml,i)
                    tmpooi<-mdl2oo(tmp.mdl,xxname_[[j]],fmlp[m])
                    colj<-cbind(colj,tmpooi[[1]]); nnj<-c(nnj,tmpooi[[2]])
                }
                tt<-rbind(tt,colj); nn<-rbind(nn,nnj)
            }
        }
    }
}
if (prn=="CX") {
    tt<-c(0,0,0,0,"Outcome",xxlbl_[[1]][-1]); nn<-c(0,0,0,0,xb[1]);
    nxl<-length(xxlbl_[[1]])-1
    for (k in (1:nclv)) {
        wdtmp0<-WD;
        if (!is.na(colvname)) {
            if (k<nclv) wdtmp0<-WD[WD[,colvname]==clv[k],];
            print(paste("Stratified by",colvname, ":", clvb[k]))
        }
        for (m in 1:nmdl) {
            for (i in (1:ny)) {
                    colj<-c(k,i,m,0,yb[i])
                    nnj <-c(k,i,m,0)
                    fml<-paste(yvname[i],"~",xvname_[1],fmlm[m]);
```

```
                    wdtmp<-removeNA(i,1,m,wdtmp0)
                    if (!is.na(colvname)) {if (k==nclv) fml<-paste(fml,"+factor(",colvname,")",sep="");}
                    if (fmlp[m]=="gam") tmp.mdl<-setgam(fml,i)
                    if (fmlp[m]=="gee") tmp.mdl<-setgee(fml,i)
                    if (fmlp[m]=="glm") tmp.mdl<-setglm(fml,i)
                    tmpooi<-mdl2oo(tmp.mdl,xxname_[[1]],fmlp[m])
                    colj<-c(colj,tmpooi[[1]][-1]); nnj<-c(nnj,tmpooi[[2]])
                    tt<-rbind(tt,colj); nn<-rbind(nn,nnj)
                }
            }
        }
    }
    if (prn=="UM") {
        tt<-c(0,0,0,0,"Exposure","Univariable","Multivariable");
    nn<-c(0,0,0,0,"Univariable","Multivariable")
        for (k in (1:nclv)) {
            wdtmp0<-WD;
            if (!is.na(colvname)) {
                if (k<nclv) wdtmp0<-WD[WD[,colvname]==clv[k],];
                print(paste("Stratified by",colvname, ":", clvb[k]))
            }
            for (i in 1:ny) {
                for (m in 1:nmdl) {
                    colm<-rep(NA,6); nnm<-rep(NA,5)
                    for (j in 1:(nx+1)) {
                        colj<-cbind(k,i,m,j,xxlbl_[[j]]); nnj<-c(k,i,m,j)
                        if    (par1==4)    {fml<-paste(yvname[i],"~",xvname_[j],fmlm[m],fmlm4[[m]][j]);}    else
    {fml<-paste(yvname[i],"~",xvname_[j],fmlm[m]);}
                        wdtmp<-removeNA(i,j,m,wdtmp0)
                        if (!is.na(colvname)) {if (k==nclv) fml<-paste(fml,"+factor(",colvname,")",sep="");}
                        if (fmlp[m]=="gam") tmp.mdl<-setgam(fml,i)
                        if (fmlp[m]=="gee") tmp.mdl<-setgee(fml,i)
                        if (fmlp[m]=="glm") tmp.mdl<-setglm(fml,i)
                        tmpooi<-mdl2oo(tmp.mdl,xxname_[[j]],fmlp[m])
                        colj<-cbind(colj,tmpooi[[1]]); nnj<-c(nnj,tmpooi[[2]])
                        if (j<=nx) {colm<-rbind(colm,colj); nnm<-rbind(nnm,nnj);
                        } else {colm<-cbind(colm[-1,],tmpooi[[1]]); nnm<-cbind(nnm[-1,],tmpooi[[2]]);}
                    }
                    tt<-rbind(tt,colm); nn<-rbind(nn,nnm)
                }
            }
        }
    }
    sink()
```

```
if (!contx & prn!="CX") rord<-c(rord[rord!=4],4)
if (prn=="X" | prn=="CX") rord<-rord[rord!=4]
if (prn=="Y") rord<-rord[rord!=2]
if (prn=="M") rord<-rord[rord!=3]
if (nx==1 & contx) rord<-rord[rord!=4]
if (nmdl==1) rord<-rord[rord!=3]
if (ny==1) rord<-rord[rord!=2]
if (is.na(colvname)) rord<-rord[rord!=1]
if (length(rord)==0) rord<-1
nrr<-length(rord)
for (i in nrr:1) {nn<-nn[order(as.numeric(nn[,rord[i]])),];tt<-tt[order(as.numeric(tt[,rord[i]])),];}
if (nn[2,4]>0) {nn[,4]<-c("Exposure",xb[as.numeric(nn[-1,4])]); } else {nn<-nn[,-4];}
if (nn[2,3]>0) {nn[,3]<-c("Model",fmlb[as.numeric(nn[-1,3])]); } else {nn<-nn[,-3];}
if (nn[2,2]>0) {nn[,2]<-c("Outcome",yb[as.numeric(nn[-1,2])]); } else {nn<-nn[,-2];}
if (!is.na(colvname)) {nn[,1]<-c(vlabel[vname==colvname],clvb_[as.numeric(nn[-1,1])]);} else
{nn<-nn[,-1];}
tb<-matrix(as.numeric(tt[,c(1:4)]),ncol=4);
if (!is.na(colvname)) {tt[,1]<-c(vlabelV[vnameV==colvname],clvb[tb[-1,1]]);}
if (ny>1) {tt[,2]<-c("Outcome",yb[tb[-1,2]]);}
if (nmdl>1) {tt[,3]<-c("Model",fmlb[tb[-1,3]]);}
nrr1<-nrr-1; oo<-tt[1,]; nc<-ncol(tt)-5; nr<-nrow(tt)
for (i in 2:nr) {
   if (nrr>1) {
      for (j in 1:nrr1) {
         if (tb[i,rord[j]]!=tb[i-1,rord[j]]) oo<-rbind(oo,c(rep(tt[i,rord[j]],5),rep(" ",nc)))
      }
   }
   oo<-rbind(oo,tt[i,])
}
if (rord[nrr]!=4 & tt[1,rord[nrr]]!="0") {oo<-cbind(oo[,rord[nrr]],oo[,-(1:5)]);} else {oo<-oo[,-(1:4)]}
w<-c(w,"</br><table border=3>", mat2htmltable(oo), "</table>")
prnopt<-c(" β  (95%CI) Pvalue / OR (95%CI) Pvalue", " β  (95%CI) Pvalue", " β +se / OR (95%CI)
*P<0.05 **P<0.01 ***P<0.001")

w<-c(w,"</br>Restults: ", prnopt[colprn])
w<-c(w,paste(c("</br>Outcome:",paste(yb,collapse="; ")),collapse=" "))
w<-c(w,paste(c("</br>Exposure:",paste(xb,collapse="; ")),collapse=" "))
for (m in 1:nmdl) w<-c(w,paste("</br>",fmlb[m],"model adjust for:", adjvb[m]))
if (smoothav>0 | smoothsv>0) w<-c(w,". </br>Generalized additive models were applied")
if (!is.na(subjvname)) w<-c(w, paste("</br>Generalized estimate equation were used, subject
ID=", subjvname, "(", gee.TYPE,")",sep=""))
w<-c(w,"</br></br>N used in the models</br><table border=3>", mat2htmltable(nn), "</table>")
w<-c(w,wd.subset)
w<-c(w,paste("</br>Created by EmpowerStats (www.empowerstats.com) and R on",Sys.Date()))
```

```
w<-c(w,"</body></html>")
fileConn<-file(paste(ofname,".htm",sep="")); writeLines(w, fileConn)
```

Supplementary Code S2. R code for generalized additive model (GAM) for the association between TNF-α and RA-ILD in female patients (Figure 2)

```
#******************** Regarding ALL Following R Code      ********************
#*** COPYRIGHT (c) 2010, 2021 X&Y Solutions, ALL RIGHT RESERVED ***********
#****************** www.EmpowerStats.com *********************************
#*************************************************************************
Sys.setlocale(category = 'LC_ALL', locale = 'English_United States.1252');
.libPaths(file.path(R.home(),'library'));
library(doBy);
options(timeout=600);
library(plotrix);
library(stringi);
library(stringr);
library(survival);
library(rms);
library(nnet);
library(car);
library(mgcv);
pdfwd<-6; pdfht<-6;
load('D:/EmpowerStats/Analysis/female/female.Rdata');
if (length(which(ls()=='EmpowerStatsR'))==0) EmpowerStatsR<-get(ls()[1]);
names(EmpowerStatsR)<-toupper(names(EmpowerStatsR));
originalVNAME<-names(EmpowerStatsR);
ofname<-'female_3_tbl';
attach(EmpowerStatsR);
sink(paste(ofname,'_datastep.lst',sep=''));
print('Creating new variable: ZV.FML');
ZV.FML<-X25+1;
summary(ZV.FML);
EmpowerStatsR<-cbind(EmpowerStatsR,ZV.FML);
print('Creating new variable: ZV.FML.LOG');
ZV.FML.LOG<- ifelse(ZV.FML>0, log(ZV.FML), NA);
summary(ZV.FML.LOG);
EmpowerStatsR<-cbind(EmpowerStatsR,ZV.FML.LOG);
rm(ZV.FML,ZV.FML.LOG);
sink();
vname<-c(NA,'X3','X12','X12.0','X12.1','X18','X23','X25','X26','ZV.FML','ZV.FML.LOG')[-1];
vlabel<-c(NA,'Age','RA-ILD','    No','    Yes','Duration','RF','TNF-a','CCP','ZV.FML','ln(TNFα )')[-1];
varused4this <- c('X3','X12','X18','X23','X25','X26','ZV.FML','ZV.FML.LOG');
pkgs<-c('mgcv','gdata');
for (g in pkgs) {
```

```
if                    (!(g                    %in%                    rownames(installed.packages())))
install.packages(g,repos='https://cloud.r-project.org');
}
library(mgcv);
library(gdata);
WD <- EmpowerStatsR; rm(EmpowerStatsR); gc();
title<-'Spline smoothing plot';
wd.subset='';
weights.var<- NA;
yvname<-c('X12');
ydist<-c('binomial');
ylink<-c('logit');
ylv<-c(2);
par1<-1;
xvname<-c('X3','X18','X23','X26');
sxf<-c(0,0,0,0);
xlv<-c(0,0,0,0);
svname<-c('ZV.FML.LOG');
sdf<-c(0);
slv<-c(0);
par3<-1;
timevar<- NA;
vname.start<- NA;
vname.stop<- NA;
subjvname<- NA;
colvname<- NA;
chk<- 1;
dec<-4;
##R package## mgcv gdata ##R package##;
vec2shift<-function(vnew,vorg,f, opt) {
   if (is.na(f[1])) {
      mean1<-mean(vorg)
      if (opt=="logit") mean1<-log(mean1/(1-mean1))
      if (opt=="log") mean1<-log(mean1)
      vnew<-vnew+(mean1-mean(vnew))
   } else {
      mean1<-tapply(vorg,factor(f),mean)
      if (opt=="logit") mean1<-log(mean1/(1-mean1))
      if (opt=="log") mean1<-log(mean1)
      mean2<-tapply(vnew,factor(f),mean);    meand<-mean1-mean2;    lvf<-levels(factor(f))
      for (z in (1:length(lvf))) {vnew[factor(f)==lvf[z]]<-vnew[factor(f)==lvf[z]]+meand[z]; }
   }
   return(vnew)
}
```

```
getNumber<-function(str, n) {
   str<-substr(str,2,nchar(str)-1)
   for (i in (1:nchar(str))) {if (substr(str,i,i)==",") {p=i; break}; }
   ifelse(n==1,return(substr(str,1,p-1)),return(substr(str,p+1,nchar(str))))
}
legLocate<-function(x,y) {
   x[is.infinite(y)]<-NA
   y[is.infinite(y)]<-NA
   xmin<-min(x,na.rm=TRUE); xmax<-max(x,na.rm=TRUE)
   ymin<-min(y,na.rm=TRUE); ymax<-max(y,na.rm=TRUE)
   yoff<-(ymax-ymin); tmp<-table(cut(x,3),cut(y,4))
   tmp.r=which.min(tmp[,4]);tmp.c=4
   if (tmp[2,1]==0) {tmp.r=2;tmp.c=1}
   if (tmp[1,1]==0) {tmp.r=1;tmp.c=1}
   if (tmp[3,1]==0) {tmp.r=3;tmp.c=1}
   if (tmp[2,4]==0) {tmp.r=2;tmp.c=4}
   if (tmp[1,4]==0) {tmp.r=1;tmp.c=4}
   if (tmp[3,4]==0) {tmp.r=3;tmp.c=4}
   pos.y<-colnames(tmp)[tmp.c];    pos.x<-rownames(tmp)[tmp.r];    pct<-0.15
   if (tmp.c==4) {
      if (min(tmp[,4])>0) {pct<-0.3}
      ymax<-ymax+yoff*pct; legy<-ymax;ymin<-ymin-yoff*0.1
   }
   if (tmp.c==1) {
      if (min(tmp[,1])>0) {pct<-0.3}
      legy<-as.numeric(getNumber(pos.y,2));ymin<-ymin-yoff*pct;ymax=ymax+yoff*0.1
   }
   legx<-as.numeric(getNumber(pos.x,1))
   return(cbind(xmin,xmax,ymin,ymax,legx,legy))
}
mat2htmltable<-function(mat) {
   t1<- apply(mat,1,function(z) paste(z,collapse="</td><td>"))
   t2<- paste("<tr><td>",t1,"</td></tr>")
   return(paste(t2,collapse=" "))
}
setgam<-function(fml,yi) {
   if (ydist[yi]=="") ydist[yi]<-"gaussian"
   if (ydist[yi]=="exact") ydist[yi]<-"binomial"
   if (ydist[yi]=="breslow") ydist[yi]<-"binomial"
   if (ydist[yi]=="gaussian") mdl<-gam(formula(fml),weights=wd$weights,data=wd,
family=gaussian(link="identity"))
   if (ydist[yi]=="binomial") mdl<-gam(formula(fml),weights=wd$weights,data=wd,
family=binomial(link="logit"))
   if (ydist[yi]=="poisson") mdl<-gam(formula(fml),weights=wd$weights,data=wd,
```

```
family=poisson(link="log"))
   if (ydist[yi]=="gamma") mdl<-gam(formula(fml),weights=wd$weights,data=wd,
family=Gamma(link="inverse"))
   if (ydist[yi]=="negbin") mdl<-gam(formula(fml),weights=wd$weights,data=wd,
family=negbin(c(1,10), link="log"))
   return(mdl)
}
gam2htmltable<-function(mdl) {
   gs<-summary(mdl)
   np<-length(gs$p.coeff)
   coe<-gs$p.table
   if (gs$family[[2]]=="log" | gs$family[[2]]=="logit") {
      cnames<-c(colnames(coe),"exp(est)","95%CI low","95%CI upp")
      coe<- cbind(coe, exp(coe[,1]), exp(coe[,1]-1.96*coe[,2]), exp(coe[,1]+1.96*coe[,2]))
   }
   if (gs$family[[2]]=="identity") {
      cnames<-c(colnames(coe),"95%CI low","95%CI upp")
      coe<- cbind(coe, coe[,1]-1.96*coe[,2], coe[,1]+1.96*coe[,2])
   }
   oo1<-cbind(c("",rownames(coe)),rbind(cnames,round(coe,dec)))
   oo<-c("</br>Linear terms effect<table border=3>",mat2htmltable(oo1),"</table>")
   if (!is.null(gs$pTerms.table)) {
      xsq<-gs$pTerms.table
      oo2<-cbind(c("",rownames(xsq)),rbind(colnames(xsq),round(xsq,dec)))
      oo<-c(oo, "</br>Chi-square tests for linear terms<table
border=3>",mat2htmltable(oo2),"</table>")
   }
   if (!is.null(gs$s.table)) {
     stb<-gs$s.table
     oo3<-cbind(c("",rownames(stb)),rbind(colnames(stb),round(stb,dec)))
     oo<-c(oo, "</br>Approximate significance of smooth terms<table
border=3>",mat2htmltable(oo3),"</table>")
   }
   p0<-c("N:", gs$n)
   p1<-c("Adj. r-square:", round(gs$r.sq,4))
   p2<-c("Deviance explained:", round(gs$dev.expl,4))
   p3<-c("UBRE score (sp.criterion):", round(gs$sp.criterion,4))
   p4<-c("Scale estimate:", gs$scale)
   p5<-c("family:", gs$family[[1]])
   p6<-c("link function:", gs$family[[2]])
   oo4<-rbind(p0,p1,p2,p3,p4,p5,p6)
   oo<-c(oo, "</br>Model statistics<table border=3>",mat2htmltable(oo4),"</table>")
   return(oo)
}
```

```
gam2pngs<-function(mdl,yi,xi) {
   pred<-predict.gam(mdl,type="terms",se.fit=TRUE)
   mfit<-NA; sfit<-NA; tmp.cname<-NA; kk0<-NA; mfit0<-NA;    ww0<-NULL
   if (xi==0) {kb=1; ke=ns;} else {kb=xi; ke=xi;}
   for (k in (kb:ke)) {
      if (slv[k]==0) {
         mfit<-cbind(mfit,apply(cbind(0,pred$fit[,sxterms[k,]]),1,sum));
         sfit<-cbind(sfit,apply(cbind(0,pred$se.fit[,sxterms[k,]]),1,sum));
         tmp.cname<-c(tmp.cname,svname[k])
         kk0<-c(kk0,k)
      }
   }
   tmp.cname<-tmp.cname[-1];    kk0<-kk0[-1]
   mfit<-matrix(mfit[,-1],ncol=length(tmp.cname));
colnames(mfit)<-paste(tmp.cname,".fit",sep="");
   sfit<-matrix(sfit[,-1],ncol=length(tmp.cname)); colnames(sfit)<-paste(tmp.cname,".se",sep="");
   if (!is.na(colvname)) {tmpfac<-wd[,colvname];} else {tmpfac<-NA;}
   if (mdl$family[2]=="logit") {
      mfit0<-mfit;    mfit0.low<-mfit0-1.96*sfit;    mfit0.upp<-mfit0+1.96*sfit;
      colnames(mfit0.low)<-paste(tmp.cname,".low",sep="");
      colnames(mfit0.upp)<-paste(tmp.cname,".upp",sep="");
      colnames(mfit0)<-paste(tmp.cname,".fit",sep="");
      ww0<-cbind(wd,mfit0); if (is.na(colvname)) ww0<-cbind(ww0,mfit0.low,mfit0.upp)
      mfit<-apply(mfit,2,function(z) vec2shift(z,mdl$fitted.value,tmpfac,"logit"))
      mfit.low<-mfit-1.96*sfit;
mfit.low<-matrix(exp(mfit.low)/(1+exp(mfit.low)),ncol=length(tmp.cname))
      mfit.upp<-mfit+1.96*sfit;
mfit.upp<-matrix(exp(mfit.upp)/(1+exp(mfit.upp)),ncol=length(tmp.cname))
      mfit<-matrix(exp(mfit)/(1+exp(mfit)),ncol=length(tmp.cname))
      colnames(mfit.low)<-paste(tmp.cname,".low",sep="");
      colnames(mfit.upp)<-paste(tmp.cname,".upp",sep="");
      colnames(mfit)<-paste(tmp.cname,".fit",sep="");
      ww<-cbind(wd,mfit); if (is.na(colvname)) ww<-cbind(ww,mfit.low,mfit.upp)
   } else if (mdl$family[2]=="log") {
      mfit0<-mfit;    mfit0.low<-mfit0-1.96*sfit;    mfit0.upp<-mfit0+1.96*sfit;
      colnames(mfit0.low)<-paste(tmp.cname,".low",sep="");
      colnames(mfit0.upp)<-paste(tmp.cname,".upp",sep="");
      colnames(mfit0)<-paste(tmp.cname,".fit",sep="");
      ww0<-cbind(wd,mfit0); if (is.na(colvname)) ww0<-cbind(ww0,mfit0.low,mfit0.upp)
      mfit<-apply(mfit,2,function(z) vec2shift(z,mdl$fitted.value,tmpfac,"log"))
      mfit.low<-mfit-1.96*sfit; mfit.low<-matrix(exp(mfit.low),ncol=length(tmp.cname))
      mfit.upp<-mfit+1.96*sfit; mfit.upp<-matrix(exp(mfit.upp),ncol=length(tmp.cname))
      mfit<-matrix(exp(mfit),ncol=length(tmp.cname))
      colnames(mfit.low)<-paste(tmp.cname,".low",sep="");
```

```
      colnames(mfit.upp)<-paste(tmp.cname,".upp",sep="");
      colnames(mfit)<-paste(tmp.cname,".fit",sep="");
      ww<-cbind(wd,mfit); if (is.na(colvname)) ww<-cbind(ww,mfit.low,mfit.upp)
    } else if (mdl$family[2]=="identity") {
      mfit<-apply(mfit,2,function(z) vec2shift(z,mdl$fitted.value,tmpfac," "))
      ww<-cbind(wd,mfit,sfit)
    } else {
      ww<-cbind(wd,mfit,sfit)
    }
    if (xi!=0) {xf<-paste(ofname,yvname[yi],svname[xi],"gam.xls",sep="_");} else
{xf<-paste(ofname,yvname[yi], "gam.xls", sep="_");}

write.table(ww,file=xf,row.names=FALSE,col.names=TRUE,sep="\t",append=FALSE,quote=FALSE)
    if (!is.null(ww0)) {
      if (xi!=0) {xf<-paste(ofname,yvname[yi],svname[xi],"gam0.xls",sep="_");} else
{xf<-paste(ofname,yvname[yi], "gam0.xls", sep="_");}

write.table(ww0,file=xf,row.names=FALSE,col.names=TRUE,sep="\t",append=FALSE,quote=FALSE
)
    }
    px<-c(20,1:9); gg<-"";
    for (k in kk0) {
       cname1<-paste(svname[k],".fit",sep="");    y.tmp<-mfit[,cname1]; y0.tmp<-NULL
       if (mdl$family[2]=="logit" | mdl$family[2]=="log") {
          cname2<-paste(svname[k],".low",sep=""); y.low<-mfit.low[,cname2]
          cname3<-paste(svname[k],".upp",sep=""); y.upp<-mfit.upp[,cname3]
          y0.tmp<-mfit0[,cname1]; y0.low<-mfit0.low[,cname2]; y0.upp<-mfit0.upp[,cname3]
       } else {
          cname2<-paste(svname[k],".se",sep=""); se.tmp<-sfit[,cname2];
          y.low<-y.tmp-1.96*se.tmp; y.upp<-y.tmp+1.96*se.tmp
       }
       x.tmp<-wd[,svname[k]];
       pngf<-paste(ofname,yvname[yi],svname[k],"smooth.png",sep="_")
       pdff<-paste(ofname,yvname[yi],svname[k],"smooth.pdf",sep="_")
       pngf0<-paste(ofname,yvname[yi],svname[k],"smooth1.png",sep="_")
       pdff0<-paste(ofname,yvname[yi],svname[k],"smooth1.pdf",sep="_")
       pngf2<-paste(ofname,yvname[yi],svname[k],"smooth2.png",sep="_")
       pdff2<-paste(ofname,yvname[yi],svname[k],"smooth2.pdf",sep="_")
       if (is.na(colvname)) {
          if (chk==1) {tmp.col<-c("red","blue");} else {tmp.col<-rep("black",2);}
          xy<-legLocate(c(x.tmp,x.tmp),c(y.low,y.upp))
          png(pngf,width=720,height=560)
          plot(y.tmp~x.tmp,ylim=c(xy[3],xy[4]),xlim=c(xy[1],xy[2]),col=tmp.col[1],type="p", pch=20,
ylab="", xlab="")
```

```
	par(new=TRUE);
	plot(y.low~x.tmp,ylim=c(xy[3],xy[4]),xlim=c(xy[1],xy[2]),col=tmp.col[2], type="p", pch=1,
ylab="", xlab="")
	par(new=TRUE);
	plot(y.upp~x.tmp,ylim=c(xy[3],xy[4]),xlim=c(xy[1],xy[2]),col=tmp.col[2], type="p", pch=1,
ylab=yb.utf8[yi], xlab=sb.utf8[k])
	dev.off()

	pdf(pdff,width=pdfwd, height=pdfht, family="GB1");
	plot(y.tmp~x.tmp,ylim=c(xy[3],xy[4]),xlim=c(xy[1],xy[2]),col=tmp.col[1],type="p", pch=20,
ylab="", xlab="")
	par(new=TRUE);
	plot(y.low~x.tmp,ylim=c(xy[3],xy[4]),xlim=c(xy[1],xy[2]),col=tmp.col[2], type="p", pch=1,
ylab="", xlab="")
	par(new=TRUE);
	plot(y.upp~x.tmp,ylim=c(xy[3],xy[4]),xlim=c(xy[1],xy[2]),col=tmp.col[2], type="p", pch=1,
ylab=yb.utf8[yi], xlab=sb.utf8[k])
	dev.off()

	png(pngf0,width=720,height=560)
	tmp.ord<-order(x.tmp);    x.tmp0<-x.tmp[tmp.ord];
	y.tmp0<-y.tmp[tmp.ord]; y.low0<-y.low[tmp.ord]; y.upp0<-y.upp[tmp.ord]
	plot(y.tmp0~x.tmp0,ylim=c(xy[3],xy[4]),xlim=c(xy[1],xy[2]),col=tmp.col[1],type="l", lty=1,
lwd=2, ylab="", xlab="")
	par(new=TRUE);
	plot(y.low0~x.tmp0,ylim=c(xy[3],xy[4]),xlim=c(xy[1],xy[2]),col=tmp.col[2], type="l", lty=3,
lwd=1, ylab="", xlab="")
	par(new=TRUE);
	plot(y.upp0~x.tmp0,ylim=c(xy[3],xy[4]),xlim=c(xy[1],xy[2]),col=tmp.col[2], type="l", lty=3,
lwd=1, ylab=yb.utf8[yi], xlab=sb.utf8[k])
	rug(x.tmp0)
	dev.off()

	pdf(pdff0,width=pdfwd, height=pdfht, family="GB1");
	tmp.ord<-order(x.tmp);    x.tmp0<-x.tmp[tmp.ord];
	y.tmp0<-y.tmp[tmp.ord]; y.low0<-y.low[tmp.ord]; y.upp0<-y.upp[tmp.ord]
	plot(y.tmp0~x.tmp0,ylim=c(xy[3],xy[4]),xlim=c(xy[1],xy[2]),col=tmp.col[1],type="l", lty=1,
lwd=2, ylab="", xlab="")
	par(new=TRUE);
	plot(y.low0~x.tmp0,ylim=c(xy[3],xy[4]),xlim=c(xy[1],xy[2]),col=tmp.col[2], type="l", lty=3,
lwd=1, ylab="", xlab="")
	par(new=TRUE);
	plot(y.upp0~x.tmp0,ylim=c(xy[3],xy[4]),xlim=c(xy[1],xy[2]),col=tmp.col[2], type="l", lty=3,
lwd=1, ylab=yb.utf8[yi], xlab=sb.utf8[k])
```

```
            rug(x.tmp0)
            dev.off()

            if (!is.null(y0.tmp)) {
               xy0<-legLocate(c(x.tmp,x.tmp),c(y0.low,y0.upp))
                  if (mdl$family[2]=="logit") {yb0<-paste("Log OR of", yb.utf8[yi]);} else
{yb0<-paste("Log RR of", yb.utf8[yi]);}
               png(pngf2,width=720,height=560)
               tmp.ord<-order(x.tmp);        x.tmp0<-x.tmp[tmp.ord];
               y0.tmp0<-y0.tmp[tmp.ord]; y0.low0<-y0.low[tmp.ord]; y0.upp0<-y0.upp[tmp.ord]

plot(y0.tmp0~x.tmp0,ylim=c(xy0[3],xy0[4]),xlim=c(xy0[1],xy0[2]),col=tmp.col[1],type="l",    lty=1,
lwd=2, ylab="", xlab="")
               par(new=TRUE);
               plot(y0.low0~x.tmp0,ylim=c(xy0[3],xy0[4]),xlim=c(xy0[1],xy0[2]),col=tmp.col[2],
type="l", lty=3, lwd=1, ylab="", xlab="")
               par(new=TRUE);
               plot(y0.upp0~x.tmp0,ylim=c(xy0[3],xy0[4]),xlim=c(xy0[1],xy0[2]),col=tmp.col[2],
type="l", lty=3, lwd=1, ylab=yb0, xlab=sb[k])
               rug(x.tmp0)
               dev.off()
            }

            rm(tmp.ord,y.tmp0,x.tmp0,y.low0,y.upp0)
            xy<-legLocate(x.tmp,wd[,1])
            pngf1<-paste(ofname,yvname[yi],svname[k],"scatter.png",sep="_")
            pdff1<-paste(ofname,yvname[yi],svname[k],"scatter.pdf",sep="_")

            png(pngf1,width=720,height=560)
            plot(y.tmp~x.tmp,ylim=c(xy[3],xy[4]),xlim=c(xy[1],xy[2]),col=tmp.col[1],           type="p",
pch=20, ylab="", xlab="")
            par(new=TRUE);
            plot(wd[,1]~x.tmp,ylim=c(xy[3],xy[4]),xlim=c(xy[1],xy[2]),type="p",pch=1,cex=0.5,
ylab=yb.utf8[yi], xlab=sb.utf8[k])
            dev.off()

            pdf(pdff1,width=pdfwd, height=pdfht, family="GB1");
            plot(y.tmp~x.tmp,ylim=c(xy[3],xy[4]),xlim=c(xy[1],xy[2]),col=tmp.col[1],           type="p",
pch=20, ylab="", xlab="")
            par(new=TRUE);
            plot(wd[,1]~x.tmp,ylim=c(xy[3],xy[4]),xlim=c(xy[1],xy[2]),type="p",pch=1,cex=0.5,
ylab=yb.utf8[yi], xlab=sb.utf8[k])
            dev.off()
```

```
        } else {
            if (chk==1) {tmp.col<-rainbow(ncg);tmp.col1<-c("red","blue")} else
{tmp.col<-rep("black",ncg);tmp.col1<-c("black","black")}
            for (b in (1:ncg)) {
               y00<-y.tmp[wd[,colvname]==colv.lv[b]]; x00<-x.tmp[wd[,colvname]==colv.lv[b]]
               xy1<-legLocate(x00,y00)

pngf1<-paste(ofname,yvname[yi],svname[k],colvname,colv.lv[b],"smooth.png",sep="_")

pdff1<-paste(ofname,yvname[yi],svname[k],colvname,colv.lv[b],"smooth.pdf",sep="_")

               png(pngf1,width=720,height=560)
               plot(y00~x00,ylim=c(xy1[3],xy1[4]),xlim=c(xy1[1],xy1[2]),col=tmp.col1[1],type="p",
pch=20, ylab=yb.utf8[yi], xlab=sb.utf8[k])
               dev.off()

               pdf(pdff1,width=pdfwd, height=pdfht, family="GB1");
               plot(y00~x00,ylim=c(xy1[3],xy1[4]),xlim=c(xy1[1],xy1[2]),col=tmp.col1[1],type="p",
pch=20, ylab=yb.utf8[yi], xlab=sb.utf8[k])
               dev.off()

            }
            xy<-legLocate(x.tmp,y.tmp)
            png(pngf,width=720,height=560)
            for (b in (1:ncg)) {
               y0<-y.tmp[wd[,colvname]==colv.lv[b]]; x0<-x.tmp[wd[,colvname]==colv.lv[b]]
               if (b>1) par(new=TRUE)
               plot(y0~x0,ylim=c(xy[3],xy[4]),xlim=c(xy[1],xy[2]),col=tmp.col[b], type="p", pch=px[b],
ylab=yb.utf8[yi], xlab=sb.utf8[k])
            }
            legend(xy[5],xy[6],colv.lb.utf8,title=colvb.utf8, pch=px[1:ncg],bty="n",col=tmp.col)
            dev.off()

            pdf(pdff,width=pdfwd, height=pdfht, family="GB1");
            for (b in (1:ncg)) {
               y0<-y.tmp[wd[,colvname]==colv.lv[b]]; x0<-x.tmp[wd[,colvname]==colv.lv[b]]
               if (b>1) par(new=TRUE)
               plot(y0~x0,ylim=c(xy[3],xy[4]),xlim=c(xy[1],xy[2]),col=tmp.col[b], type="p", pch=px[b],
ylab=yb[yi], xlab=sb[k])
            }
            legend(xy[5],xy[6],colv.lb.utf8,title=colvb.utf8, pch=px[1:ncg],bty="n",col=tmp.col)
            dev.off()

            png(pngf0,width=720,height=560)
```

```
            for (b in (1:ncg)) {
                y0<-y.tmp[wd[,colvname]==colv.lv[b]]; x0<-x.tmp[wd[,colvname]==colv.lv[b]]
                tmp.ord<-order(x0); x00<-x0[tmp.ord]; y00<-y0[tmp.ord];
                if (b>1) par(new=TRUE)
                plot(y00~x00,ylim=c(xy[3],xy[4]),xlim=c(xy[1],xy[2]),col=tmp.col[b],    type="l",    lty=b,
lwd=2, ylab=yb.utf8[yi], xlab=sb.utf8[k])
                rm(tmp.ord,x00,y00)
            }
            legend(xy[5],xy[6],colv.lb.utf8,title=colvb.utf8,lty=(1:ncg),bty="n",col=tmp.col)
            dev.off()

            pdf(pdff0,width=pdfwd, height=pdfht, family="GB1");
            for (b in (1:ncg)) {
                y0<-y.tmp[wd[,colvname]==colv.lv[b]]; x0<-x.tmp[wd[,colvname]==colv.lv[b]]
                tmp.ord<-order(x0); x00<-x0[tmp.ord]; y00<-y0[tmp.ord];
                if (b>1) par(new=TRUE)
                plot(y00~x00,ylim=c(xy[3],xy[4]),xlim=c(xy[1],xy[2]),col=tmp.col[b],    type="l",    lty=b,
lwd=2, ylab=yb.utf8[yi], xlab=sb.utf8[k])
                rm(tmp.ord,x00,y00)
            }
            legend(xy[5],xy[6],colv.lb.utf8,title=colvb.utf8,lty=(1:ncg),bty="n",col=tmp.col)
            dev.off()

            if (!is.null(y0.tmp)) {
                xy0<-legLocate(c(x.tmp,x.tmp),c(y0.low,y0.upp))
                    if    (mdl$family[2]=="logit")    {yb0<-paste("Log    OR    of",    yb.utf8[yi]);}    else
{yb0<-paste("Log RR of", yb.utf8[yi]);}
                png(pngf2,width=720,height=560)
                for (b in (1:ncg)) {
                    y0<-y0.tmp[wd[,colvname]==colv.lv[b]]; x0<-x.tmp[wd[,colvname]==colv.lv[b]]
                    tmp.ord<-order(x0); x00<-x0[tmp.ord];    y00<-y0[tmp.ord];
                    if (b>1) par(new=TRUE)
                    plot(y00~x00,ylim=c(xy0[3],xy0[4]),xlim=c(xy[1],xy[2]),col=tmp.col[b],        type="l",
lty=b, lwd=2, ylab=yb0, xlab=sb.utf8[k])
                    rm(tmp.ord,x00,y00)
                }
                legend(xy0[5],xy0[6],colv.lb.utf8,title=colvb.utf8,lty=(1:ncg),bty="n",col=tmp.col)
                dev.off()
            }

        }
        gg<-c(gg,paste("<td>",yb[yi],"  vs.  ",sb[k],"</br><a  href=\"",pngf,"\"  target=_BLANK><img
src=\"",pngf,"?time=\" width=320,height=320></a></td>",sep=""))
    }
```

```
    return(gg)
}
adjmean<-function(mdl, yi, xi) {
    if (length(xvname)>0) {allvname<- c(xvname,svname); all.lv<-c(xlv,slv);} else
{allvname<-c(svname); all.lv<-slv;}
    if (!is.na(colvname)) allvname<-c(allvname,colvname)
    nv = length(allvname)
    xi.lv <- levels(factor(WD[,svname[xi]]))
    newd0 <- matrix(0,ncol=nv,nrow=length(xi.lv))
    colnames(newd0)<-allvname
    for (b in 1:length(all.lv)) {
        if (all.lv[b]==0) {
            newd0[,b]<-mean(WD[,allvname[b]],na.rm=TRUE)
        } else {
            uniqv <- unique(WD[,allvname[b]])
            newd0[,b]<-uniqv[which.max(tabulate(match(WD[,allvname[b]], uniqv)))]
        }
    }
    newd0[,svname[xi]]<-as.numeric(xi.lv)
    if (!is.na(colvname)) {
        for (b in 1:ncg) {
            newd1<-newd0; newd1[,colvname]<-as.numeric(colv.lv[b]);
            if (b==1) {newD<-newd1;} else {newD<-rbind(newD,newd1);}
        }
        f<-table(wd[,svname[xi]],wd[,colvname])
    } else {
        newD<-newd0; ncg<-1;
        f<-table(wd[,svname[xi]])
    }
    pred<-predict(mdl, data.frame(newD),se.fit=TRUE)
    meany.pop0<-tapply(wd[,yvname[yi]],wd[,svname[xi]],function(z) mean(z,na.rm=TRUE))[1]
    if (ylink[yi]=="logit") meany.pop0<-log(meany.pop0/(1-meany.pop0));
    if (ylink[yi]=="log") meany.pop0<-log(meany.pop0);
    shift<-meany.pop0-pred$fit[1]
    y.fit <- pred$fit+shift
    y.low <- pred$fit+shift-pred$se.fit*1.96
    y.upp <- pred$fit+shift+pred$se.fit*1.96
    y.pred<- cbind(y.fit,y.low,y.upp);
    tmp.ylab<-paste("Mean of", yb.utf8[yi]);
    cname.pred<-c("Mean","Mean.low","Mean.upp")
    if (ylink[yi]=="logit") {
        y.pred<-exp(y.pred); y.pred<-y.pred/(1+y.pred);
        tmp.ylab<-paste("% of", yb.utf8[yi]);
        cname.pred<-c("Rate","Rate.low","Rate.upp")
```

```
    }
    if (ylink[yi]=="log") {
        y.pred<-exp(y.pred); tmp.ylab<-paste("% of", yb.utf8[yi]);
        cname.pred<-c("Rate","Rate.low","Rate.upp")
    }
    y.fit<-y.pred[,1]; y.low<-y.pred[,2]; y.upp<-y.pred[,3]
    if (length(xvname)>0) {
        tmp.ylab<-paste("Adjusted", tmp.ylab)
        cname.pred<-paste("adj.", cname.pred, sep="")
    }
    y.pred<-round(y.pred,dec)
    colnames(y.pred)<-cname.pred
    y.pred <-cbind(newD[,svname[xi]],y.pred); colnames(y.pred)[1]<-svname[xi]
    if (!is.na(colvname)) {
        y.pred<-cbind(newD[,colvname],y.pred); colnames(y.pred)[1]<-colvname
    }
    y.pred<-rbind(colnames(y.pred),y.pred)
    px<-c(20,1:9)
    if (chk==1) {tmp.col<-rainbow(ncg);tmp.col1<-c("red","blue");
    } else {tmp.col<-rep("black",ncg);tmp.col1<-tmp.col}

    pngf<-paste(ofname,yvname[yi],svname[xi],"adjmean.png",sep="_")
    pdff<-paste(ofname,yvname[yi],svname[xi],"adjmean.pdf",sep="_")

    if (ncg>1) {
        xy<-legLocate(newD[,svname[xi]],c(y.fit))
        png(pngf,width=720,height=560)
        for (b in 1:ncg) {
            x.tmp<-newD[newD[,colvname]==colv.lv[b],svname[xi]]
            y.tmp<-y.fit[newD[,colvname]==colv.lv[b]]
            if (b>1) par(new=TRUE)
            plot(y.tmp~x.tmp,ylim=c(xy[3],xy[4]),xlim=c(xy[1],xy[2]),col=tmp.col[b],type="b",
pch=px[b],
                ylab=tmp.ylab, xlab=sb.utf8[xi])
        }
        legend(xy[5],xy[6],colv.lb,title=colvb,pch=px[1:ncg],bty="n",col=tmp.col)
        dev.off()

        pdf(pdff,width=pdfwd, height=pdfht, family="GB1");
        for (b in 1:ncg) {
            x.tmp<-newD[newD[,colvname]==colv.lv[b],svname[xi]]
            y.tmp<-y.fit[newD[,colvname]==colv.lv[b]]
            if (b>1) par(new=TRUE)
            plot(y.tmp~x.tmp,ylim=c(xy[3],xy[4]),xlim=c(xy[1],xy[2]),col=tmp.col[b],type="b",
```

```
pch=px[b],
        ylab=tmp.ylab, xlab=sb.utf8[xi])
    }
    legend(xy[5],xy[6],colv.lb,title=colvb,pch=px[1:ncg],bty="n",col=tmp.col)
    dev.off()

    for (b in 1:ncg) {
      x.tmp<-newD[newD[,colvname]==colv.lv[b],svname[xi]]
      y.tmp<-y.fit[newD[,colvname]==colv.lv[b]]
      y.lci<-y.low[newD[,colvname]==colv.lv[b]]
      y.uci<-y.upp[newD[,colvname]==colv.lv[b]]
      xy<-legLocate(c(x.tmp,x.tmp),c(y.lci,y.uci))

png(paste(ofname,yvname[yi],svname[xi],colvname,colv.lv[b],"CI.png",sep="_"),width=720,heigh
t=560)
      plotCI(x.tmp,y=y.tmp,li=y.lci,ui=y.uci,pch=20,lwd=1,col=tmp.col[1],
xlim=c(xy[1],xy[2]),ylim=c(xy[3],xy[4]),
        ylab=tmp.ylab, xlab=sb.utf8[xi], main=paste(colvb.utf8, colv.lb.utf8[b],sep=": "))
      lines(x.tmp,y.tmp,lty=2)
      dev.off()


pdf(paste(ofname,yvname[yi],svname[xi],colvname,colv.lv[b],"CI.pdf",sep="_"),width=pdfwd,
height=pdfht, family="GB1");
      plotCI(x.tmp,y=y.tmp,li=y.lci,ui=y.uci,pch=20,lwd=1,col=tmp.col[1],
xlim=c(xy[1],xy[2]),ylim=c(xy[3],xy[4]),
        ylab=tmp.ylab, xlab=sb.utf8[xi], main=paste(colvb.utf8, colv.lb.utf8[b],sep=": "))
      lines(x.tmp,y.tmp,lty=2)
      dev.off()

    }
  } else {
    x.tmp<-newD[,svname[xi]]
    xy<-legLocate(c(x.tmp,x.tmp),c(y.low,y.upp))
    png(pngf,width=720,height=560)
    plotCI(x.tmp,y=y.fit,li=y.low,ui=y.upp,pch=20,lwd=1,col=tmp.col[1],
xlim=c(xy[1],xy[2]),ylim=c(xy[3],xy[4]),
        ylab=tmp.ylab, xlab=sb.utf8[xi], main="Adjusted mean & 95% CI")
    lines(x.tmp,y.fit,lty=2)
    dev.off()

    pdf(pdff,width=pdfwd, height=pdfht, family="GB1");
    plotCI(x.tmp,y=y.fit,li=y.low,ui=y.upp,pch=20,lwd=1,col=tmp.col[1],
xlim=c(xy[1],xy[2]),ylim=c(xy[3],xy[4]),
```

```
            ylab=tmp.ylab, xlab=sb.utf8[xi], main="Adjusted mean & 95% CI")
        lines(x.tmp,y.fit,lty=2)
        dev.off()
    }
    oo<-c("</br>Adjusted mean ",yb[yi], " by ", sb[xi], "<table border=3>",mat2htmltable(y.pred),"</table>")
    gg<-paste("<td>",yb[yi]," vs. ",sb[xi],"</br><a href=\"",pngf,"\" target=_BLANK><img src=\"",pngf,"?time=\" width=320,height=320></a></td>", sep="")
    return(list(oo,gg))
}
if (!is.na(weights.var)) {weights<-WD[,weights.var];} else {weights<-1;}
WD<-cbind(WD,weights);
vlabelN<-(substr(vlabel,1,1)==" ");
vlabelZ<-vlabel[vlabelN];vlabelV<-vlabel[!vlabelN]
vnameV<-vname[!vlabelN];vnameZ<-vname[vlabelN]
ny<-length(yvname); yb<-vlabelV[match(yvname,vnameV)]; yb[is.na(yb)]<-yvname[is.na(yb)]
ns<-length(svname); sb<-vlabelV[match(svname,vnameV)]; sb[is.na(sb)]<-svname[is.na(sb)]
yb.utf8<-yb; Encoding(yb.utf8)<-"UTF-8"
sb.utf8<-sb; Encoding(sb.utf8)<-"UTF-8"
ssf<-rep(",fx=FALSE", ns); ssf[sdf>0]<-paste(",k=",sdf[sdf>0],sep="")
sxStr<-paste("s(",svname,ssf,sep="")
sxStr[slv>0]<-paste("factor(",svname[slv>0],")",sep="")
sxx<-paste("s(",svname,")",sep="")
sxx[slv>0]<-paste("factor(",svname[slv>0],")",sep="")
sxx<-matrix(sxx,ncol=1)
if (!is.na(colvname)) {
    sxStr[slv==0]<-paste(sxStr[slv==0],",by=factor(", colvname, ")",sep="")
    sxStr[slv>0]<-paste(sxStr[slv>0],"*factor(", colvname, ")",sep="")
    colv.lv<-levels(factor(WD[,colvname])); ncg<-length(colv.lv); colvb<-vlabel[vname==colvname];
    colv.lb<-vlabelZ[match(paste(colvname,colv.lv,sep="."),vnameZ)]
    colv.lb[is.na(colv.lb)]<-colv.lv[is.na(colv.lb)]
    colvb<-vlabelV[match(colvname,vnameV)]; if (is.na(colvb)) colvb<-colvname;
    colvb.utf8<-colvb; Encoding(colvb.utf8)<-"UTF-8"; colv.lb.utf8<-colv.lb; Encoding(colv.lb.utf8)<-"UTF-8"
    sxplots<-NA; sxterms<-NA
    for (i in (1:ns)) {
        sxplots<-c(sxplots,paste(svname[i],"_",colvname,colv.lv,sep=""));
        sxterms<-rbind(sxterms,paste(sxx[i,],":factor(",colvname,")",colv.lv,sep=""))
    }
    sxplots<-sxplots[-1]; sxterms<-matrix(sxterms[-1,],ncol=ncg)
    sxterms<-cbind(paste("factor(",colvname,")",sep=""),sxterms)
} else {ncg<-1;sxplots<-svname; sxterms<-sxx;}
sxStr[slv==0]<-paste(sxStr[slv==0],")",sep="")
nx<-0
```

```
if (length(xvname)>0) {
   if (!is.na(colvname)) {xvname<-xvname[xvname!=colvname];}
   nx<-length(xvname);
}
if (nx>0) {
   xb<-vlabelV[match(xvname,vnameV)]; xb[is.na(xb)]<-xvname[is.na(xb)];
   xvv<-xvname; xvv[xlv>2]<-paste("factor(",xvname[xlv>2],")",sep="")
   if (!is.na(colvname)) {
      xvv[sxf=="S" | sxf=="s"]<-paste("factor(",colvname,")*",xvv[sxf=="S" | sxf=="s"],sep="")
   }
   xv1<-paste(xvv,collapse="+")
}
if (is.na(par1)) par1<-1
if (ny!=ns & par1==2) par1<-1
if (par1==3) {nterms=ns*ncg*15+nx;} else {nterms=ncg*15+nx;}
w<-c("<!DOCTYPE html><html lang='zh'><head><meta charset='utf-8'></head><body>")
w<-c(w,paste("<h2>", title, "</h2>"))
wtab<-"</br></br>Generalize additive models</br>"
wpng<-"</br><table>";
for (i in (1:ny)) {
   if (par1!=3) {
      wtmp<-"";
      if (par1==2) {jstart<-i; jstop<-i;} else {jstart<-1; jstop<-ns;}
      for (j in (jstart:jstop)) {
         tmp.xx<-c(yvname[i],svname[j])
         if (nx>0) tmp.xx<-c(tmp.xx,xvname)
         if (!is.na(colvname[1])) tmp.xx<-c(tmp.xx,colvname)
         wd<-WD[,tmp.xx];
         wd<-wd[apply(is.na(wd),1,sum)==0,]
         fml<-paste(yvname[i],"~",sxStr[j],sep="")
         if (!is.na(colvname)) fml<-paste(fml,"+factor(",colvname,")",sep="")
         if (nx>0) fml<-paste(fml,"+",xv1,sep="")
         tmp.gam<-setgam(fml,i)
         wtab<-c(wtab,paste("</br></br>Outcome:",yb[i]))
         wtab<-c(wtab,paste("</br>Exposure:",sb[j]))
         wtab<-c(wtab,gam2htmltable(tmp.gam))
         if (slv[j]==0) {
            wtmp<-c(wtmp,gam2pngs(tmp.gam,i,j))
         } else {
            stmp<-adjmean(tmp.gam,i,j)
            wtmp<-c(wtmp,stmp[[2]])
            wtab<-c(wtab,stmp[[1]])
         }
      }
```

```
        wpng<-c(wpng,"<tr>",wtmp,"</tr>")
    } else {
            tmp.xx<-c(yvname[i],svname);
            if (nx>0) tmp.xx<-c(tmp.xx,xvname)
            if (!is.na(colvname[1])) tmp.xx<-c(tmp.xx,colvname)
            wd<-WD[,tmp.xx];
            wd<-wd[apply(is.na(wd),1,sum)==0,]
            fml<-paste(yvname[i],"~",paste(sxStr,collapse="+"),sep="")
            if (!is.na(colvname)) fml<-paste(fml,"+factor(",colvname,")",sep="")
            if (nx>0) fml<-paste(fml,"+",xv1,sep="")
            tmp.gam<-setgam(fml,i)
            wtab<-c(wtab,paste("</br></br>Outcome:",yb[i]))
            wtab<-c(wtab,gam2htmltable(tmp.gam))
            if (sum(slv==0)>0) {
                wpng<-c(wpng,"<tr>",gam2pngs(tmp.gam,i,0),"</tr>")
            }
            if (sum(slv>0)>0) {
                for (k in 1:ns) {
                    if (slv[k]>0) {
                        stmp<-adjmean(tmp.gam,i,k)
                        wtab<-c(wtab,stmp[[1]])
                        wpng<-c(wpng,stmp[[2]])
                    }
                }
            }
    }
}
wpng<-c(wpng,"</table>")
w<-c(w,wpng,wtab)
w<-c(w,wd.subset)
w<-c(w,paste("</br>Created by EmpowerStats (www.empowerstats.com) and R on",Sys.Date()))
w<-c(w,"</body></html>")
fileConn<-file(paste(ofname,".htm",sep="")); writeLines(w, fileConn)
```

Supplementary Code S3. R code for two-piecewise logistic regression for the association between TNF-α and RA-ILD in female patients (Table 4)

```
#******************** Regarding ALL Following R Code       ********************
#*** COPYRIGHT (c) 2010, 2021 X&Y Solutions, ALL RIGHT RESERVED ***********
#******************* www.EmpowerStats.com **********************************
#****************************************************************************
Sys.setlocale(category = 'LC_ALL', locale = 'English_United States.1252');
.libPaths(file.path(R.home(),'library'));
library(doBy);
options(timeout=600);
```

```
library(plotrix);
library(stringi);
library(stringr);
library(survival);
library(rms);
library(nnet);
library(car);
library(mgcv);
pdfwd<-6; pdfht<-6;
load('D:/EmpowerStats/Analysis/female/female.Rdata');
if (length(which(ls()=='EmpowerStatsR'))==0) EmpowerStatsR<-get(ls()[1]);
names(EmpowerStatsR)<-toupper(names(EmpowerStatsR));
originalVNAME<-names(EmpowerStatsR);
ofname<-'female_2_tbl';
attach(EmpowerStatsR);
sink(paste(ofname,'_datastep.lst',sep=''));
print('Creating new variable: ZV.FML');
ZV.FML<-X25+1;
summary(ZV.FML);
EmpowerStatsR<-cbind(EmpowerStatsR,ZV.FML);
print('Creating new variable: ZV.FML.LOG');
ZV.FML.LOG<- ifelse(ZV.FML>0, log(ZV.FML), NA);
summary(ZV.FML.LOG);
EmpowerStatsR<-cbind(EmpowerStatsR,ZV.FML.LOG);
rm(ZV.FML,ZV.FML.LOG);
sink();
vname<-c(NA,'X3','X12','X12.0','X12.1','X18','X23','X25','X26','ZV.FML','ZV.FML.LOG')[-1];
vlabel<-c(NA,'Age','RA-ILD','    No','    Yes','Duration','RF','TNF-a','CCP','ZV.FML','ln(TNFα)')[-1];
varused4this <- c('X3','X12','X18','X23','X25','X26','ZV.FML','ZV.FML.LOG');
pkgs<-c('MASS','gdata','geepack','mgcv');
for (g in pkgs) {
if (!(g %in% rownames(installed.packages())))
install.packages(g,repos='https://cloud.r-project.org');
}
library(MASS);
library(gdata);
library(geepack);
library(mgcv);
WD <- EmpowerStatsR; rm(EmpowerStatsR); gc();
title<-'Threshold analysis';
wd.subset='';
weights.var<- NA;
yvname<-c('X12');
ydist<-c('binomial');
```

```
ylink<-c('logit');
ylv<-c(2);
par1<-'Automatic search';
avname<-c('X3','X18','X23','X26');
saf<-c(0,0,0,0);
alv<-c(0,0,0,0);
bvar<- NA;
xvname<-c('ZV.FML.LOG');
xlv<-c(0);
chk<- 0;
cox<- 0;
timevar<- NA;
vname.start<- NA;
subjvname<- NA;
gee.TYPE<-c('independence', 'unstructured', 'exchangeable', 'AR1')[0];
prn<-1;
dec<-2;
##R package## MASS gdata geepack mgcv ##R package##;
pvformat<-function(p,dec) {
  pp <- sprintf(paste("%.",dec,"f",sep=""),as.numeric(p))
  if (is.matrix(p)) {pp<-matrix(pp, nrow=nrow(p));
colnames(pp)<-colnames(p);rownames(pp)<-rownames(p);}
  lw <- paste("<",substr("0.00000000000",1,dec+1),"1",sep="");
  pp[as.numeric(p)<(1/10^dec)]<-lw
  return(pp)
}
numfmt<-function(p,dec) {
  if (is.list(p)) p<-as.matrix(p)
  pp <- sprintf(paste("%.",dec,"f",sep=""),as.numeric(p))
  if (is.matrix(p)) {pp<-matrix(pp,
nrow=nrow(p));colnames(pp)<-colnames(p);rownames(pp)<-rownames(p);}
  pp[as.numeric(p)>10000000]<- "inf."
  pp[is.na(p) | gsub(" ","",p)==""]<- ""
  pp[p=="-Inf"]<-"-Inf"
  pp[p=="Inf"]<-"Inf"
  return(pp)
}
mat2htmltable<-function(mat) {
  t1<- apply(mat,1,function(z) paste(z,collapse="</td><td>"))
  t2<- paste("<tr><td>",t1,"</td></tr>")
  return(paste(t2,collapse=" "))
}
setgam<-function(fml,yi,wdtmp) {
  if (ydist[yi]=="") ydist[yi]<-"gaussian"
```

```
  if (ydist[yi]=="exact") ydist[yi]<-"binomial"
  if (ydist[yi]=="breslow") ydist[yi]<-"binomial"
  if (ydist[yi]=="gaussian") mdl<-try(gam(formula(fml),weights=wdtmp$weights,data=wdtmp,
family=gaussian(link="identity")))
  if (ydist[yi]=="binomial") mdl<-try(gam(formula(fml),weights=wdtmp$weights,data=wdtmp,
family=binomial(link="logit")))
  if (ydist[yi]=="poisson") mdl<-try(gam(formula(fml),weights=wdtmp$weights,data=wdtmp,
family=poisson(link="log")))
  if (ydist[yi]=="gamma") mdl<-try(gam(formula(fml),weights=wdtmp$weights,data=wdtmp,
family=Gamma(link="inverse")))
  if (ydist[yi]=="negbin") mdl<-try(gam(formula(fml),weights=wdtmp$weights,data=wdtmp,
family=negbin(c(1,10), link="log")))
  return(mdl)
}
setgee<-function(fml,yi, wdtmp) {
  if (ydist[yi]=="") ydist[yi]<-"gaussian"
  if (ydist[yi]=="exact") ydist[yi]<-"binomial"
  if (ydist[yi]=="breslow") ydist[yi]<-"binomial"
  if (ydist[yi]=="gaussian")
md<-try(geeglm(formula(fml),id=wdtmp[,subjvname],corstr=gee.TYPE,family="gaussian",weights
=wdtmp$weights,data=wdtmp))
  if (ydist[yi]=="binomial")
md<-try(geeglm(formula(fml),id=wdtmp[,subjvname],corstr=gee.TYPE,family="binomial",weights
=wdtmp$weights,data=wdtmp))
  if (ydist[yi]=="poisson")
md<-try(geeglm(formula(fml),id=wdtmp[,subjvname],corstr=gee.TYPE,family="poisson",weights=
wdtmp$weights,data=wdtmp))
  if (ydist[yi]=="gamma")
md<-try(geeglm(formula(fml),id=wdtmp[,subjvname],corstr=gee.TYPE,family="Gamma",weights=
wdtmp$weights,data=wdtmp))
  if (ydist[yi]=="negbin")
md<-try(geeglm.nb(formula(fml),id=wdtmp[,subjvname],corstr=gee.TYPE,weights=wdtmp$weigh
ts,data=wdtmp))
  return(md)
}
setglm<-function(fml,yi, wdtmp) {
  if (ydist[yi]=="") ydist[yi]<-"gaussian"
  if (ydist[yi]=="exact") ydist[yi]<-"binomial"
  if (ydist[yi]=="breslow") ydist[yi]<-"binomial"
  if (ydist[yi]=="gaussian")
md<-try(glm(formula(fml),family="gaussian",weights=wdtmp$weights,data=wdtmp))
  if (ydist[yi]=="binomial")
md<-try(glm(formula(fml),family="binomial",weights=wdtmp$weights,data=wdtmp))
  if (ydist[yi]=="poisson")
```

```
md<-try(glm(formula(fml),family="poisson",weights=wdtmp$weights,data=wdtmp))
  if (ydist[yi]=="gamma")
md<-try(glm(formula(fml),family="Gamma",weights=wdtmp$weights,data=wdtmp))
  if (ydist[yi]=="negbin") md<-try(glm.nb(formula(fml),weights=wdtmp$weights,data=wdtmp))
  return(md)
}
mdl2oo<-function(mdl, xxname, opt) {
  if (is.na(mdl[[1]][1])) return(rep(" ",times=length(xxname)))
  if (substr(mdl[[1]][1],1,5)=="Error") return(rep(" ",times=length(xxname)))
  decp<-dec+2; if (decp>4) decp<-4
  gs<-summary(mdl); print(mdl$formula); print(gs)
  if (opt=="gam") {gsparm <- gs$p.table; } else {gsparm <- gs$coefficients;}
  gsp<-gsparm[match(xxname,rownames(gsparm)),]
  if (length(xxname)==1) {beta<-gsp[1]; se<-gsp[2]; pv<-gsp[4];
  } else {beta<-gsp[,1]; se<-gsp[,2]; pv<-gsp[,4]; }
  ci1<- beta-1.96*se; ci2<- beta+1.96*se
  pvx<-substr(rep("****",length(pv)),1,(pv<=0.05)+(pv<=0.01)+(pv<=0.001))
  if (colprn==3) {pvv<-pvx;} else {pvv<-pvformat(pv,decp);}
  if ((colprn!=2) & (gs$family[[2]]=="log" | gs$family[[2]]=="logit")) {
    o1<-paste(numfmt(exp(beta),dec)," (",numfmt(exp(ci1),dec),",
",numfmt(exp(ci2),dec),")",sep="")
  } else {
    if (colprn<3) {o1<-paste(numfmt(beta,dec), " (",numfmt(ci1,dec),",
",numfmt(ci2,dec),")",sep="")
    } else {o1<-paste(numfmt(beta,dec), "+",numfmt(se,dec),sep="");}
  }
  o1<-paste(o1,pvv); o1[is.na(beta)]<-NA
  return(o1)
}
removeNA<-function(i,j,wdf) {
 vvv<-c(yvname[i],xvname[j],avname,subjvname,bvar,vname.start,timevar);
 vvv<-vvv[!is.na(vvv)]; vvv<-vvv[vvv>" "]
 tmp<-is.na(wdf[,vvv]);
 return(wdf[apply(tmp,1,sum)==0,])
}
get.tpval<-function(i,j,g,opt,wdtmp, tppmin=NA, tppmax=NA) {
  if (sum(is.na(wdtmp))>0) {
    if (is.na(g)) {wdtmp<-removeNA(i,j,WD);
    } else if (g<nblv) {wdtmp0<-WD[WD[,bvar]==blv[g],]; wdtmp<-removeNA(i,j,wdtmp0);
    } else {wdtmp<-removeNA(i,j,WD); }
  }
  if (is.na(g)) {fmladj1<-fmladj;
  } else if (g<nblv) {fmladj1<-fmladj;
  } else {fmladj1<-paste(fmladj,"+factor(",bvar,")",sep="");}
```

```
xTMP <- wdtmp[,xvname[j]]
tmp.ss<-seq(0.05,0.95,0.05)
tp<-quantile(xTMP,probs=tmp.ss,na.rm=TRUE)
tmp.llk<-rep(NA,length(tmp.ss))
fml<-paste(yvname[i],"~",xvname[j],"+tmp.X",fmladj1)
if (!is.na(tppmin) & !is.na(tppmax)) {
   tp2.min = tppmin; tp2.max = tppmax;
} else {
   for (k in (1:length(tmp.ss))) {
      tmp.X<-(xTMP > tp[k])*(xTMP-tp[k]); wdtmp1<-cbind(wdtmp,tmp.X)
      if (opt=="glm" | opt=="gee") tmp.mdl<-setglm(fml, i, wdtmp1);
      if (opt=="gam") tmp.mdl<-setgam(fml, i, wdtmp1);
      tmp.llk[k]<-logLik(tmp.mdl)
      rm(wdtmp1, tmp.X)
   }
   tp1<-tmp.ss[which.max(tmp.llk)]
   tp2.min = tp1 - 0.04
   tp2.max = tp1 + 0.04
   if (tp2.min<0.05) {tp2.min=0.05}
   if (tp2.max>0.95) {tp2.max=0.95}
}
tp.pctlrange<-quantile(xTMP,probs=c(tp2.min,tp2.max),na.rm=TRUE)
tp.range<-unique(xTMP[xTMP>tp.pctlrange[1] & xTMP<tp.pctlrange[2]])
while (length(tp.range)>5) {
   tmp.pct3<-quantile(tp.range,probs=c(0,0.25,0.5,0.75,1),type=3)
   tmp.llk3<-rep(NA,3)
   for (k in (2:4)) {
      tmp.X<-(xTMP>tmp.pct3[k])*(xTMP-tmp.pct3[k]); wdtmp1<-cbind(wdtmp,tmp.X)
      if (opt=="glm" | opt=="gee") tmp.mdl<-setglm(fml, i, wdtmp1);
      if (opt=="gam") tmp.mdl<-setgam(fml, i, wdtmp1);
      tmp.llk3[k-1]<-logLik(tmp.mdl)
      rm(wdtmp1, tmp.X)
     }
     tmp.min3<-which.max(tmp.llk3)
     tp.range<-tp.range[tp.range>=tmp.pct3[tmp.min3] & tp.range<=tmp.pct3[tmp.min3+2]]
}
if (length(tp.range)>0) {
   if (length(tp.range)==1) {tp.val=tp.range[1];} else {
      tmp.llk<-rep(NA,length(tp.range))
      for (k in (1:length(tp.range))) {
         tmp.X<-(xTMP>tp.range[k])*(xTMP-tp.range[k]); wdtmp1<-cbind(wdtmp,tmp.X)
         if (opt=="glm" | opt=="gee") tmp.mdl<-setglm(fml, i, wdtmp1);
         if (opt=="gam") tmp.mdl<-setgam(fml, i, wdtmp1);
         tmp.llk[k]<-logLik(tmp.mdl)
```

```
            rm(wdtmp1, tmp.X)
        }
        tp.val<-tp.range[which.max(tmp.llk)]
      }
  } else { tp.val<-tp.pctlrange[1];}
  return(round(tp.val,dec));
}
get2lines<-function(i,j,g,tp.value,opt) {
  if (is.na(g)) {fmladj1<-fmladj;wdtmp<-removeNA(i,j,WD);
  } else if (g<nblv)
{fmladj1<-fmladj;wdtmp0<-WD[WD[,bvar]==blv[g],];wdtmp<-removeNA(i,j,wdtmp0);
  } else {fmladj1<-paste(fmladj,"+factor(",bvar,")",sep="");wdtmp<-removeNA(i,j,WD);}
  xTMP<-wdtmp[,xvname[j]]
  tmp.X1<-(xTMP<=tp.value)*(xTMP-tp.value)
  tmp.X2<-(xTMP> tp.value)*(xTMP-tp.value)
  wdtmp1<-cbind(wdtmp,xTMP,tmp.X1,tmp.X2)
  fml0<-paste(yvname[i],"~xTMP+tmp.X2",fmladj1)
  fml1<-paste(yvname[i],"~tmp.X1+tmp.X2",fmladj1)
  fml2<-paste(yvname[i],"~xTMP",fmladj1)
  fmlp<-paste(yvname[i],"~xTMP+tmp.X2")
  tmpn<-nrow(wdtmp)
  print(paste("tp.value ==", tp.value))
  if (opt=="glm") {
      tmp.mdl0<-setglm(fml0,i,wdtmp1); tmp.mdl1<-setglm(fml1,i,wdtmp1)
      tmp.mdl2<-setglm(fml2,i,wdtmp1); tmp.mdlp<-setglm(fmlp,i,wdtmp1)
  }
  if (opt=="gam") {
      tmp.mdl0<-setgam(fml0,i,wdtmp1); tmp.mdl1<-setgam(fml1,i,wdtmp1)
      tmp.mdl2<-setgam(fml2,i,wdtmp1); tmp.mdlp<-setgam(fmlp,i,wdtmp1)
  }
  if (opt=="gee") {
      tmp.mdl0<-setgee(fml0,i,wdtmp1); tmp.mdl1<-setgee(fml1,i,wdtmp1)
      tmp.mdl2<-setgee(fml2,i,wdtmp1); tmp.mdlp<-setglm(fmlp,i,wdtmp1)
  }
  pd<-predict(tmp.mdlp,data.frame(xTMP=tp.value,tmp.X2=0), se.fit=TRUE)
  prd<-paste(numfmt(pd$fit,dec),"          (",numfmt(pd$fit-1.96*pd$se.fit,dec),",          ",
numfmt(pd$fit+1.96*pd$se.fit, dec),")",sep="")
  m2<-mdl2oo(tmp.mdl2,"xTMP",opt)
  m1<-mdl2oo(tmp.mdl1,c("tmp.X1","tmp.X2"),opt)
  m0<-mdl2oo(tmp.mdl0,"tmp.X2",opt)
  if (opt=="gee") {
      plrt<-try(anova(tmp.mdl0,tmp.mdl2)$"P(>|Chi|)",TRUE)
      plrt<-ifelse((plrt<="9" && plrt>="0"), pvformat(plrt,3),"-")
  } else {plrt<- pvformat(1-pchisq(2*(logLik(tmp.mdl0)[1]-logLik(tmp.mdl2)[1]),1),3);}
```

```
   plrti<-""; plrtx<-""
   if (!is.na(g) & g==nblv) {
      fmli<-paste(yvname[i],"~xTMP+xTMP*factor(",bvar,")",fmladj)

fmlx<-paste(yvname[i],"~tmp.X1+factor(",bvar,")*tmp.X1+tmp.X2+factor(",bvar,")*tmp.X2",fmlad
j)
      if (opt=="gee") {
         tmp.mdli<-setgee(fmli,i,wdtmp1)
         tmp.mdlx<-setgee(fmlx,i,wdtmp1)
      } else {
         if (opt=="glm") {tmp.mdli<-setglm(fmli,i,wdtmp1);tmp.mdlx<-setglm(fmlx,i,wdtmp1);}
         if (opt=="gam") {tmp.mdli<-setgam(fmli,i,wdtmp1);tmp.mdlx<-setgam(fmlx,i,wdtmp1);}
         plrti<- paste("P-interaction:",
pvformat(1-pchisq(2*(logLik(tmp.mdli)[1]-logLik(tmp.mdl2)[1]),nblv-2),3));
         plrtx<- paste("P-interaction:",
pvformat(1-pchisq(2*(logLik(tmp.mdlx)[1]-logLik(tmp.mdl1)[1]),2*(nblv-2)),3));
      }
      print(summary(tmp.mdli));
      print("Interaction model === >")
      print(paste("tp.value ==", tp.value))
      print(summary(tmp.mdlx))
   }
   oo<-list(c(plrti,m2,plrtx,tp.value,m1,m0,prd,plrt),tmpn)
   return(oo)
}
get3lines<-function(i,j,g,tp.value,opt) {
   if (is.na(g)) {fmladj1<-fmladj;wdtmp<-removeNA(i,j,WD);
   } else if (g<nblv)
{fmladj1<-fmladj;wdtmp0<-WD[WD[,bvar]==blv[g],];wdtmp<-removeNA(i,j,wdtmp0);
   } else {fmladj1<-paste(fmladj,"+factor(",bvar,")",sep="");wdtmp<-removeNA(i,j,WD);}
   xTMP<-wdtmp[,xvname[j]]; tp1<-tp.value[1]; tp2<-tp.value[2]
   tmp.X1<- (xTMP< tp1)*(xTMP-tp1)
   tmp.X2<-((xTMP>=tp1) & (xTMP<=tp2))*(xTMP-tp1)
   tmp.X3<- (xTMP> tp2)*(xTMP-tp2)
   tmp.B1<- (xTMP>tp2)
   wdtmp1<-cbind(wdtmp,xTMP,tmp.X1,tmp.X2,tmp.X3,tmp.B1)
   fml0<-paste(yvname[i],"~xTMP+tmp.X1+tmp.X3+tmp.B1",fmladj1)
   fml1<-paste(yvname[i],"~tmp.X1+tmp.X2+tmp.X3+tmp.B1",fmladj1)
   fml2<-paste(yvname[i],"~xTMP",fmladj1)
   tmpn<-nrow(wdtmp)
   print(paste("tp.value ==", tp.value))
   if (opt=="glm") {
      tmp.mdl0<-setglm(fml0,i,wdtmp1); tmp.mdl1<-setglm(fml1,i,wdtmp1);
tmp.mdl2<-setglm(fml2,i,wdtmp1);
```

```
  }
  if (opt=="gam") {
      tmp.mdl0<-setgam(fml0,i,wdtmp1);                    tmp.mdl1<-setgam(fml1,i,wdtmp1);
tmp.mdl2<-setgam(fml2,i,wdtmp1);
  }
  if (opt=="gee") {
      tmp.mdl0<-setgee(fml0,i,wdtmp1);                    tmp.mdl1<-setgee(fml1,i,wdtmp1);
tmp.mdl2<-setgee(fml2,i,wdtmp1);
  }
  m2<-mdl2oo(tmp.mdl2,"xTMP",opt)
  m1<-mdl2oo(tmp.mdl1,c("tmp.X1","tmp.X2","tmp.X3"),opt)
  m0<-mdl2oo(tmp.mdl0,c("tmp.X1","tmp.X3"),opt)
  if (opt=="gee") {
      plrt<-try(anova(tmp.mdl0,tmp.mdl2)$"P(>|Chi|)",TRUE)
      plrt<-ifelse((plrt<="9" && plrt>="0"), pvformat(plrt,3),"-")
  } else {plrt<- pvformat(1-pchisq(2*(logLik(tmp.mdl0)[1]-logLik(tmp.mdl2)[1]),1),3);}
  plrti<-""; plrtx<-""
  if (!is.na(g) & g==nblv) {
    fmli<-paste(yvname[i],"~xTMP+xTMP*factor(",bvar,")",fmladj)

fmlx<-paste(yvname[i],"~tmp.X1+factor(",bvar,")*tmp.X1+tmp.X2+factor(",bvar,")*tmp.X2+tmp.X
3+factor(",bvar,")*tmp.X3+tmp.B1",fmladj)
    if (opt=="gee") {
       tmp.mdli<-setgee(fmli,i,wdtmp1)
       tmp.mdlx<-setgee(fmlx,i,wdtmp1)
    } else {
       if (opt=="glm") {tmp.mdli<-setglm(fmli,i,wdtmp1);tmp.mdlx<-setglm(fmlx,i,wdtmp1);}
       if (opt=="gam") {tmp.mdli<-setgam(fmli,i,wdtmp1);tmp.mdlx<-setgam(fmlx,i,wdtmp1);}
       plrti<-                                                        paste("P-interaction:",
pvformat(1-pchisq(2*(logLik(tmp.mdli)[1]-logLik(tmp.mdl2)[1]),nblv-2),3));
       plrtx<-                                                        paste("P-interaction:",
pvformat(1-pchisq(2*(logLik(tmp.mdlx)[1]-logLik(tmp.mdl1)[1]),3*(nblv-2)),3));
    }
    print(summary(tmp.mdli));
    print("Interaction model === >")
    print(paste("tp.value ==", tp.value))
    print(summary(tmp.mdlx))
  }
  oo<-list(c(plrti,m2,plrtx,paste(tp.value,collapse=", "),m1,m0,plrt),tmpn)
  return(oo)
}
getci4tp<-function(i,j,g,opt,tp0=NA) {
  set.seed(123456)
  if (is.na(g)) {wdt<-removeNA(i,j,WD);
```

```
    } else if (g<nblv) {wdt<-WD[WD[,bvar]==blv[g],];wdt<-removeNA(i,j,wdt);
    } else {wdt<-removeNA(i,j,WD);}
    nnwd<-nrow(wdt); tp.vv<-rep(NA,1000)
    if (!is.na(tp0)) {
        tpp0 = sum(wdt[,xvname[j]] < tp0)/length(wdt[,xvname[j]])
        tppmin = max(tpp0 - 0.09, 0.05)
        tppmax = min(tpp0 + 0.09, 0.95)
    } else {
        tppmin = NA; tppmax = NA
    }
    for (s in (1:1000)) {
        WDi<-wdt[sample(1:nnwd,nnwd,replace=T),]
        tp.vv[s]<-get.tpval(i, j, NA, opt, WDi, tppmin, tppmax); rm(WDi)
    }
    tpci<-paste(quantile(tp.vv,probs=c(0.025,0.975)),collapse=", ")
    return(tpci);
}
if (!is.na(weights.var)) {weights<-WD[,weights.var];} else {weights<-1;}
WD<-cbind(WD,weights);
vlabelN<-(substr(vlabel,1,1)==" ");
vlabelZ<-vlabel[vlabelN];vlabelV<-vlabel[!vlabelN]
vnameV<-vname[!vlabelN];vnameZ<-vname[vlabelN];
allvname<-c(yvname,xvname,bvar,avname,subjvname,vname.start,timevar,"weights");
allvname<-allvname[!is.na(allvname)]
WD<-WD[,allvname];
w<-c("<!DOCTYPE html><html lang='zh'><head><meta charset='utf-8'></head><body>")
w<-c(w,paste("<h2>", title, "</h2>"))
if (length(avname)>0) {
    if (sum((saf=="s" | saf=="S") & alv>0)>0) w<-c(w,"</br>Spline smoothing only applies for
continuous variables")
    if (!is.na(subjvname)) saf<-rep(0,length(saf))
}
if (sum(xlv>0)>0) w<-c(w,"Categorical exposure variables were ignored")
xvname<-xvname[xlv==0];
if (!is.na(subjvname)) WD<-WD[order(WD[,subjvname]),];

fmladj<-""; avb=""; smoothav<-0;
if (length(avname)>0) {
    avb<-vlabelV[match(avname,vnameV)];
    avname_ <- avname
    smoothavi<-((saf=="s" | saf=="S") & alv==0)
    smoothav<-sum(smoothavi)
    avname_[smoothavi]<-paste("s(",avname[smoothavi],")",sep="")
    avb1<-avb
```

```
    avb1[smoothavi]<-paste(avb[smoothavi],"(Smooth)",sep="")
    avname_[alv>0]<-paste("factor(",avname[alv>0],")",sep="")
    fmladj<-paste("+",paste(avname_,collapse="+"))
}
if (is.na(bvar)) {
    blvb<-"N"; blvb_<-"N"; nblv<-1; blbl<-"";
} else {
    blbl<-vlabelV[match(bvar,vnameV)]; if (is.na(blbl)) blbl<-bvar;
    blv<-levels(factor(WD[,bvar])); nblv<-length(blv)+1
    blvb_<-vlabelZ[match(paste(bvar,".",blv,sep=""),vnameZ)];
    blvb_[is.na(blvb_)]<-blv[is.na(blvb_)];
    blvb<-c(paste(blbl,blvb_,sep="="),"Total");
    blvb_<-c(blvb_,"Total")
    WD<-WD[!is.na(WD[,bvar]),]
}
ny=length(yvname); nx=length(xvname);
xb<-vlabelV[match(xvname,vnameV)]; xb[is.na(xb)]<-xvname[is.na(xb)]
yb<-vlabelV[match(yvname,vnameV)]; yb[is.na(yb)]<-yvname[is.na(yb)]
opt<-ifelse(!is.na(subjvname), "gee", ifelse(smoothav>0, "gam", "glm")) ;
colprn<-prn
if (is.na(par1)) par1<-"";
if (is.numeric(par1)) {tp.vv<-par1;
} else {
    tmp<-as.numeric(strsplit(gsub(",", " ",par1)," ")[[1]]);    tp.vv<-c(tmp[!is.na(tmp)],NA)
}
prn<-ifelse(!is.na(bvar), "S", ifelse(nx>1 & ny==1, "X", "Y"));
if (length(tp.vv)>2) tp.vv<-tp.vv[1:2]
ntp<-length(tp.vv);
getci<-FALSE
prnopt<-c(" β  (95%CI) Pvalue / OR (95%CI) Pvalue", " β  (95%CI) Pvalue", " β +se / OR (95%CI)
*P<0.05 **P<0.01 ***P<0.001")


if (ntp==1) {
    cc0<-c("Model I","?? One line effect");
    cc0<-c(cc0,"Model II","?? Turning point(K)","?? < K effect 1","?? > K effect 2","?? effect 2 - 1")
    cc0<-c(cc0,"?? Model fit value at K")
    if (is.na(tp.vv[1]) & chk==1) getci<-TRUE;
} else {
    cc0<-c("Model I","?? One line effect");
    cc0<-c(cc0,"Model II","?? Turing points(K1,K2)","?? < K1 effect 1","?? K1-K2 effect 2","?? > K2
effect 3")
    cc0<-c(cc0,"?? effect 1 - 2","?? effect 3 - 2")
}
```

```
if (opt=="gee") {cc0<-c(cc0,"ANOVA compare 2 models");} else {cc0<-c(cc0,"LRT test");}
if (getci) cc0<-c(cc0,"95% CI of TP(K)");
sink(paste(ofname,".lst",sep=""))
nn<-c("Outcome","Exposure",blvb);
if (prn=="Y") {
   for (j in 1:nx) {
      tt<-cc0;
      for (i in 1:ny) {
         if (is.na(tp.vv[1])) {tp.v<-get.tpval(i,j,NA,opt,NA);} else {tp.v<-tp.vv;}
         if (ntp==1) tmpij<-get2lines(i,j,NA,tp.v,opt);
         if (ntp==2) tmpij<-get3lines(i,j,NA,tp.v,opt);
         if (getci) {tt<-cbind(tt,c(tmpij[[1]],getci4tp(i,j,NA,opt,tp.v)));} else {tt<-cbind(tt,tmpij[[1]]);}
         nn<-rbind(nn,c(yb[i],xb[j],tmpij[[2]]))
      }
      tt<-rbind(c("Outcome: ",yb),tt)
      w<-c(w,paste("</br>For exposure:",xb[j]))
      w<-c(w,"</br><table border=3>", mat2htmltable(tt), "</table>")
   }
}
if (prn=="X") {
   for (i in 1:ny) {
      tt<-cc0;
      for (j in 1:nx) {
         if (is.na(tp.vv[1])) {tp.v<-get.tpval(i,j,NA,opt,NA);} else {tp.v<-tp.vv;}
         if (ntp==1) tmpij<-get2lines(i,j,NA,tp.v,opt);
         if (ntp==2) tmpij<-get3lines(i,j,NA,tp.v,opt);
         if (getci) {tt<-cbind(tt,c(tmpij[[1]],getci4tp(i,j,NA,opt,tp.v)));} else {tt<-cbind(tt,tmpij[[1]]);}
         nn<-rbind(nn,c(yb[i],xb[j],tmpij[[2]]))
      }
      tt<-rbind(c("Exposure: ",xb),tt)
      w<-c(w,paste("</br>For outcome:",yb[i]))
      w<-c(w,"</br><table border=3>", mat2htmltable(tt), "</table>")
   }
}
if (prn=="S") {
   for (i in 1:ny) {
      tt<-cc0;
      for (j in 1:nx) {
         nnij<-c(yb[i],xb[j])
         for (g in 1:nblv) {
            if (is.na(tp.vv[1])) {tp.v<-get.tpval(i,j,g,opt,NA);} else {tp.v<-tp.vv;}
            if (ntp==1) tmpij<-get2lines(i,j,g,tp.v,opt);
            if (ntp==2) tmpij<-get3lines(i,j,g,tp.v,opt);
            if (getci) {tt<-cbind(tt,c(tmpij[[1]],getci4tp(i,j,g,opt,tp.v)));} else {tt<-cbind(tt,tmpij[[1]]);}
```

```r
                nnij<-c(nnij,tmpij[[2]])
            }
            nn<-rbind(nn,nnij)
        }
        tt<-rbind(c(blbl,blvb_),tt)
        w<-c(w,paste("</br>For outcome:",yb[i]))
        w<-c(w,paste("</br>For Exposure:",xb[j]))
        w<-c(w,"</br><table border=3>", mat2htmltable(tt), "</table>")
    }
}
sink()
w<-c(w,"</br>Results in table:", prnopt[colprn])
w<-c(w,paste(c("</br>Outcome:",paste(yb,collapse="; ")),collapse=" "))
w<-c(w,paste(c("</br>Exposure:",paste(xb,collapse="; ")),collapse=" "))
if (length(avname)==0) avb1<-"None";
w<-c(w,paste(c("</br>Adjust for:",paste(avb1,collapse="; ")),collapse=" "))
if (smoothav>0) w<-c(w,". Generalized additive models were applied")
if (opt=="gee") w<-c(w, paste("</br>Generalized estimate equation were used, subject ID=", subjvname, "(", gee.TYPE,")",sep=""))
w<-c(w,"</br></br>N used in the models</br><table border=3>", mat2htmltable(nn), "</table>")
w<-c(w,wd.subset)
w<-c(w,paste("</br>Created by EmpowerStats (www.empowerstats.com) and R on",Sys.Date()))
w<-c(w,"</body></html>")
fileConn<-file(paste(ofname,".htm",sep="")); writeLines(w, fileConn)
```
